# Integrating single‐cell and spatial transcriptomes reveals *COL4A1/2* facilitates the spatial organisation of stromal cells differentiation in breast phyllodes tumours

**DOI:** 10.1002/ctm2.1611

**Published:** 2024-03-14

**Authors:** Xia Li, Xuewen Yu, Jiaxin Bi, Xu Jiang, Lu Zhang, Zhixin Li, Mumin Shao

**Affiliations:** ^1^ Department of Pathology Shenzhen Traditional Chinese Medicine Hospital Shenzhen P.R. China; ^2^ Department of Pathology The Fourth Clinical Medical College of Guangzhou University of Chinese Medicine Shenzhen P.R. China; ^3^ Department of Surgery Shenzhen Traditional Chinese Medicine Hospital Shenzhen P.R. China; ^4^ Department of Surgery The Fourth Clinical Medical College of Guangzhou University of Chinese Medicine Shenzhen P.R. China

**Keywords:** breast phyllodes tumour heterogeneity, fibroepithelial lesions, single‐cell RNA sequencing, spatial transcriptome, tumour grading

## Abstract

**Background:**

Breast phyllodes tumours (PTs) are a unique type of fibroepithelial neoplasms with metastatic potential and recurrence tendency. However, the precise nature of heterogeneity in breast PTs remains poorly understood. This study aimed to elucidate the cell subpopulations composition and spatial structure and investigate diagnostic markers in the pathogenesis of PTs.

**Methods:**

We applied single‐cell RNA sequencing and spatial transcriptomes on tumours and adjacent normal tissues for integration analysis. Immunofluorescence experiments were conducted to verify the tissue distribution of cells. Tumour cells from patients with PTs were cultured to validate the function of genes. To validate the heterogeneity, the epithelial and stromal components of tumour tissues were separated using laser capture microdissection, and microproteomics data were obtained using data‐independent acquisition mass spectrometry. The diagnostic value of genes was assessed using immunohistochemistry staining.

**Results:**

Tumour stromal cells harboured seven subpopulations. Among them, a population of widely distributed cancer‐associated fibroblast‐like stroma cells exhibited strong communications with epithelial progenitors which underwent a mesenchymal transition. We identified two stromal subpopulations sharing epithelial progenitors and mesenchymal markers. They were inferred to further differentiate into transcriptionally active stromal subpopulations continuously expressing *COL4A1/2*. The binding of *COL4A1/2* with *ITGA1/B1* facilitated a growth pattern from the stroma towards the surrounding glands. Furthermore, we found consistent transcriptional changes between intratumoural heterogeneity and inter‐patient heterogeneity by performing microproteomics studies on 30 samples from 11 PTs. The immunohistochemical assessment of 97 independent cohorts identified that *COL4A1/2* and *CSRP1* could aid in accurate diagnosis and grading.

**Conclusions:**

Our study demonstrates that *COL4A1/2* shapes the spatial structure of stromal cell differentiation and has important clinical implications for accurate diagnosis of breast PTs.

## Graphical abstract

1. We revealed breast PT's stromal cell subpopulations and differentiation hierarchy.

2. High expression of *COL4A1/2* accompanied stromal cell differentiation.

3. Binding of *COL4A1/2* and *ITGA1/B1* facilitated the spatial structure of stromal cells.

4. A large sample validated that *COL4A1/2* can aid accurate diagnosis and grading of breast PTs.

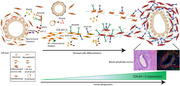

## 1 BACKGROUND

Fibroepithelial lesions of the breast are a heterogeneous group of neoplasms that can be classified as fibroadenomas and phyllodes tumours (PTs). Fibroadenomas can be safely managed and many of them do not require routine excision. In contrast, PTs have a potential for recurrence and metastasis.^1^ In addition, they are particularly prevalent in young Asian women.^2^ Morphologically, PTs present a typical leaf‐like pattern containing both stromal and epithelial components. The World Health Organization grades breast PTs as benign (∼60%–75%), borderline (∼15%–20%) or malignant (∼10%–20%) based on histologic features.^3^ Surgical resection is the main treatment for breast PTs; however, some excised tumours with negative margins still have local recurrence and even metastasis.^4^ Recurrence is positively associated with tumour malignancy, with benign, borderline and malignant recurrence rates of 6%–9%, 11%–16% and 14%–21%, respectively.^5^ Metastases can occur in the lungs, bones, pleura and pancreas, which may have fatal effects on patients.^6^, ^7^, ^8^ Furthermore, morphologically similar tumour cells can exhibit different behaviours, outcomes and responses to treatment. The lack of understanding of the inherent heterogeneity of breast PTs renders the assessment of malignant behaviour and growth trend of tumour cells in clinical practice difficult.^9^, ^10^, ^11^, ^12^ This hinders accurate diagnosis and even leads to the possibility of recurrence and metastasis. Therefore, a comprehensive exploration of the cellular and molecular bases of breast PTs will allow us to better manage this disease.

Breast PTs exhibit mutations,^13^, ^14^, ^15^ with variations in different regions of tumour tissues.^16^, ^17^ In addition, changes in gene expression or protein product levels could better reflect the intrinsic pathogenesis of breast PTs.^18^, ^19^, ^20^ Some studies have reported that macrophages could stimulate myofibroblast differentiation, and interactions between them promoted the proliferation of malignant tumour cells.^21^, ^22^ This suggests that specific cell populations exist in breast PTs tissue that contribute to tumour cell progression. Despite these observations, the precise nature of heterogeneity in breast PTs, encompassing genetic changes and their role in the acquisition of the malignant behaviour of tumour cells, remains poorly understood.

To address this gap, this study aimed to elucidate the cell subpopulations’ composition and spatial structure and investigate diagnostic markers in the pathogenesis of PTs. We used single‐cell RNA sequencing (scRNA‐seq) to investigate samples of untreated breast PTs from patients with both borderline and malignant forms. We explored the cellular subpopulations, their differentiation and intercellular relationships. By combining spatial transcriptome (ST) data from the same patient, we described the spatial distribution of cell subpopulations and revealed their spatial growth patterns. Furthermore, we investigated the critical genes underlying cellular differentiation and validated gene function using tumour cells from patients with PTs. Finally, using laser capture microdissection (LCM)‐based microproteome data, immunofluorescence (IF), transmission electron microscopy (TEM) and immunohistochemistry (IHC) on additional 108 independent breast PTs, we validated our results and assessed the diagnostic value of markers that could be used to grade patients.

## 2 METHODS

### 2.1 Collection of clinical human patient samples

**FIGURE 1:**
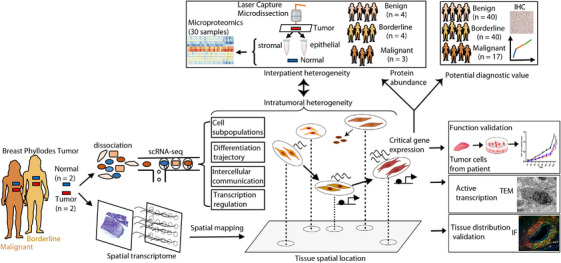
Framework of the study design. We applied single‐cell RNA sequencing (scRNA‐seq) and spatial transcriptome (ST) on fresh tumour and adjacent normal tissues of one borderline and one malignant breast phyllodes tumour (PT) for integration analysis. We resolved cellular subpopulations and inferred differentiation trajectory and intercellular communication between subpopulations. Accompanying cellular differentiation, we revealed critical gene expression and underlying transcriptional regulation. By integrating ST data, we showed that critical genes facilitated subpopulations in shaping tissue structures. Leveraging microproteome data, immunofluorescence (IF), transmission electron microscopy (TEM), immunohistochemistry (IHC) and in vitro experiments, we performed validations and assessed the potential diagnostic value of the critical gene in large‐scale patient samples. Created with BioRender.com.

This study was approved by the Institutional Review Board of Shenzhen Traditional Chinese Medicine Hospital (ethics number: K2022‐020‐02). The experimental and analytical framework of this study is illustrated in Figure 1. Human breast PTs and adjacent normal tissues from a patient with borderline PT (P1) and a patient with malignant PT (P2), prior to any local or systemic therapy, were obtained with informed consent. Immediately after surgical resection, the tissues were dissected to isolate the tumour and adjacent normal regions for single‐cell dissociation. Adjacent normal tissue was sampled at a distance of at least 2 cm from the tumour tissue. Fresh tissues were maintained on ice in Roswell Park Memorial Institute (RPMI) 1640 medium containing 10% fetal bovine serum (FBS). All diagnoses were confirmed by a histological review by two pathologists. The experimental details of tissue dissociation for scRNA‐seq and sample preparation for ST are described in the Supporting Information.

### 2.2 scRNA‐seq

Cell suspensions were freshly prepared according to the manufacturer's protocol of 10× Chromium 3′ v3.1 kit (10× Genomics). Library preparation and sequencing, using PE150, were conducted on an Illumina NovaSeq 6000 platform (Illumina, Inc.). Tumour tissues from P1 and P2 were scheduled for scRNA‐seq processing at different times because of challenges in securing fresh tissues from different patients simultaneously.

### 2.3 ST library preparation and sequencing

The Visium Spatial Gene Expression Slide & Reagent kit (10× Genomics) was used to construct the sequencing library according to the manufacturer's user guide (CG000239, 10× Genomics). A 10‐µm frozen tissue section was placed on one of the Visium Spatial Gene Expression Slides (1000186, 10× Genomics), specifically on the Capture Areas. After staining the tissue with haematoxylin and eosin, bright‐field images were captured following the spatial transcriptomics procedure. Tissue permeabilisation was performed for the optimal time as established in the above tissue optimisation procedures. A reverse transcription experiment was then conducted, and a sequencing library was prepared following the manufacturer's protocol.

Sequencing was performed using the NovaSeq PE150 platform (Illumina, Inc.) according to the manufacturer's instructions, at an average depth of 300 million read pairs per sample.

### 2.4 scRNA‐seq data pre‐processing

Raw base call (BCL) files (binary files with raw sequencing data generated from Illumina sequencers) were converted to the FASTQ format using mkfastq with 10× Genomics Cell Ranger (version 4.0.0). Reads were aligned with the human genome (GRCh38‐1.2.0) using the STAR algorithm (version 2.5.1b) with default settings.^23^ The ‘count’ command of Cell Ranger was used to generate raw gene‐barcode matrices. The pre‐processing steps including filtration, quality control and normalisation are included in the Supporting Information.

### 2.5 Cell clustering, identity determination and function characterisation

The non‐negative matrix factorisation (NMF) algorithm^24^ was employed to cluster cells, and each cluster was annotated by their expression of cell marker genes (see Supporting Information). To evaluate the consistency of cell identity with well‐known functions, the signatures of each cluster were subjected to functional enrichment analysis using clusterProfiler (version 4.2.2)^25^ R package. The *p*‐value was adjusted using the Benjamini–Hochberg method. The FindMarkers function in Seurat was used to compare differentially expressed genes between the two cell groups. Genes were defined as significantly differentially expressed between the two groups when the adjusted *p*‐value was less than .01.

Copy number variation (CNV) analysis based on scRNA‐seq data was performed using the infercnv (version 1.8.1)^26^ R package, to estimate tumour cells. Immune and epithelial cells were used as reference cells. Genes were sorted according to their chromosomal location, and expression was centered at 1.

### 2.6 scRNA‐seq data downstream analysis

For cell differentiation trajectory construction, cellular communication inference and transcriptional regulatory relationship analysis, see the Supporting Information.

### 2.7 ST data analysis

The raw Visium sequencing reads and histological images of ST were quality checked and processed using Space Ranger (version spaceranger‐1.2.0, 10× Genomics) with the commands SpaceRanger, mkfastq and spaceranger count. The filtered gene spot count matrix output and fiducially aligned image data were further analysed using Seurat. Spots were filtered for more than 20% of the reads that mapped to the mitochondrial genome, and the SCTransform function in Seurat was used for data transformation. Clustering of spots was performed using NMF based on the top 5000 variable genes, similar to NMF clustering in the scRNA‐seq data. The signatures of each region were extracted using the same process as that used for the scRNA‐seq data.

### 2.8 Cell type deconvolution

The relative abundance of single‐cell clusters in each of the visible spots was inferred using the SPOTlight^27^ algorithm based on seeded NMF regression. As multiple cell types may overlap in each spot, we determined the proportion of each scRNA‐seq cluster in each spot. Only the cell types that contributed to at least 9% of the spot signature were calculated. Alternatively, we applied the FindTransferAnchors function in the Seurat package to check the spatial mapping of single‐cell types on the ST data.

### 2.9 Signature enrichment

A total of 29 sets of genes representing the tumour microenvironment gene expression signatures were collected from a previous study.^28^ We performed signature enrichment analysis for each of these 29 gene sets in all cell subpopulations. Only those gene sets, such as cancer‐associated fibroblasts (CAF), epithelial–mesenchymal transition (EMT) and matrix, that displaying dominant enrichment variability among stromal cell subpopulations, were used to assess the potential function of the subpopulations. The phosphatidylinositol 3‐kinase (PI3K)–protein kinase B (Akt), mitogen‐activated protein kinase (MAPK) pathway genes and hypoxia signature were downloaded from MSigDB.^29^ Signature enrichment scoring across cell clusters and spots was performed using the AddModuleScore function in Seurat with 25 bins and 100 control genes.

### 2.10 Stromal and epithelial components isolation using LCM

Tumour and adjacent normal tissue samples were collected from four benign, four borderline and three malignant PTs from the same hospital as mentioned above. Samples from benign and borderline tumours were further dissected to isolate tissues responsive to stromal and epithelial components using LCM (Leica LMD6500; Leica Microsystems CMS GmbH). In total, 30 samples were obtained from 11 patients, including eight epithelial (sourced from four benign and four borderline patients), eight stromal (sourced from four benign and four borderline patients), three malignant tumours and 11 adjacent normal tissues (sourced from four benign, four borderline and three malignant patients).

### 2.11 Microproteomic data generation and analysis

We extracted proteins from the LCM tissues and used data‐independent acquisition (DDA) mass spectrometry to generate microproteomic data (Supporting Information). MaxQuant (version 1.6.0.1)^30^ was used to perform a database search against human SwissProt entries in the UniProtKB database (Uniprot, release 2018_02) for protein identification and quantification. The following parameters were used: minimal peptide length of seven amino acids, fixed modification of cysteine carbamidomethylation and variable modification of methionine oxidation and N‐terminal protein acetylation. A false‐positive control was performed with a false discovery rate of 1% for peptide or protein identification.

The cluster analysis was performed using NMF on the 500 most variable proteins. The clusters were set from 2 to 10, and 100 runs were performed for each cluster to obtain consensus results. From the clustering outcomes, we selected three major clusters that represented the structure of the samples. The signature proteins of each cluster were extracted using the extractFeatures function, based on the maximum contribution to the classification. The R package GSVA (version 1.42.0)^31^ was used for the enrichment analysis of signature proteins and single‐cell type signature genes in each sample. The clusterProfiler package (version 4.2.2)^25^ was used to perform the functional analysis of signature proteins.

### 2.12 IHC, IF and TEM

For IHC, IF and TEM experiments and procedures on breast PT tissues, see the Supporting Information.

### 2.13 Knockdown experiments in human breast PTs tumour cells

We obtained primary human breast PT tumour cell culture using tumour tissues from a borderline breast PT and established immortalised cells through SV40T lentiviral infection (Supporting Information). To knock down *COL4A1* and *COL4A2* in cells, two independent shRNA sequences were designed and cloned into the pLent‐U6‐Puro vector. Another shRNA with a nonsense sequence was used as the negative control. Immortalised primary PT tumour cells were inoculated into 24‐well plates and transfected 24 h later using Lipo2000 reagent (Invitrogen) at a 70% fusion rate. The transfection mixture, composed of 1 µg plasmid and 2 µL Lipo2000 with 50 µL Opti‐MEM (Gibco) was incubated for 10 min before being added to the cells. The solution was changed after 12 h. The shRNA sequences were designed as follows:

*COL4A1*‐shRNA1: CTGTTGGGCCTCCAGGATTTATTCAAGAGATAAATCCTGGAGGCCCAACAGTTTTTT
*COL4A1*‐shRNA2: TGGCCCACCTGGAATTGTTATTTCAAGAGAATAACAATTCCAGGTGGGCCATTTTTT
*COL4A2*‐shRNA1: CAGGAAGCCCTGGATTTAAAGTTCAAGAGACTTTAAATCCAGGGCTTCCTGTTTTTT
*COL4A2*‐shRNA2: CCAGGAATGAAAGACATTAAATTCAAGAGATTTAATGTCTTTCATTCCTGGTTTTTT

### 2.14 Cell proliferation assay

Cell proliferation assays were conducted using a cell counting kit‐8 (CCK‐8; DOJINDO). We seeded 5000 cells per well in a 96‐well plate, and following a 12 h incubation, 10 µL of CCK‐8 solution was added to the cells. Absorbance at 450 nm was measured after 1 h of incubation and used to plot the cell growth curve.

### 2.15 RNA extraction and reverse transcription quantitative polymerase chain reaction (RT‐qPCR)

Total RNA was extracted from the cells or tissue samples using a TRIzol kit (Invitrogen). Subsequently, 1 µg of RNA was immediately reverse‐transcribed into cDNA using the TransScript one‐step gDNA removal and cDNA synthesis SuperMix (Transgen). The qPCR was performed using iTaq Universal SYBR Green SMX 500 (Bio‐Rad) on an ABI 7500 real‐time PCR instrument (Applied Biosystems). Each experiment was replicated three times, and *GAPDH* was used as an internal reference gene. The data were obtained during the extension process. The primer sequences used for RT‐qPCR were as follows:

SV40T‐F: ATTGCCTGGAACGCAGTGAG
SV40T‐R: GCAAACTCAGCCACAGGTCT
*GAPDH*‐F: AAATCAAGTGGGGCGATGCTG
*GAPDH*‐R: GCAGAGATGATGACCCTTTTG
*COL4A1*‐F: GGGATGCTGTTGAAAGGTGAA
*COL4A1*‐R: GGTGGTCCGGTAAATCCTGG
*COL4A2*‐F: AAGGGCTTCATCGGAGACC
*COL4A2*‐R: CCAGCGTCACCTTTCCACC

### 2.16 Statistical analysis

The comparison of gene expression levels obtained by RT‐qPCR between different groups and the differences in optical density (OD) values between different transfection groups of cells were analysed by GraphPad Prism (RRID: SCR_002798, version 7.04) software. Results were presented as mean ± standard deviation (*n* = 3). A two‐way analysis of variance, followed by Tukey's multiple comparison test, was performed to compare differences between groups. Differences were considered statistically significant at *p* < .05 (^*^ *p* < .05, ^**^ *p* < .01, ^***^ *p* < .001, ^****^ *p* < .0001); otherwise, they were deemed insignificant. Comparisons of IHC results between groups were analysed using SPSS (RRID: SCR_002865, version 16.0) software with the chi‐square test or Fisher's exact test. *p* < .05 was considered statistically significant.

## 3 RESULTS

### 3.1 scRNA‐seq determines tumour stromal cell subpopulations

**FIGURE 2:**
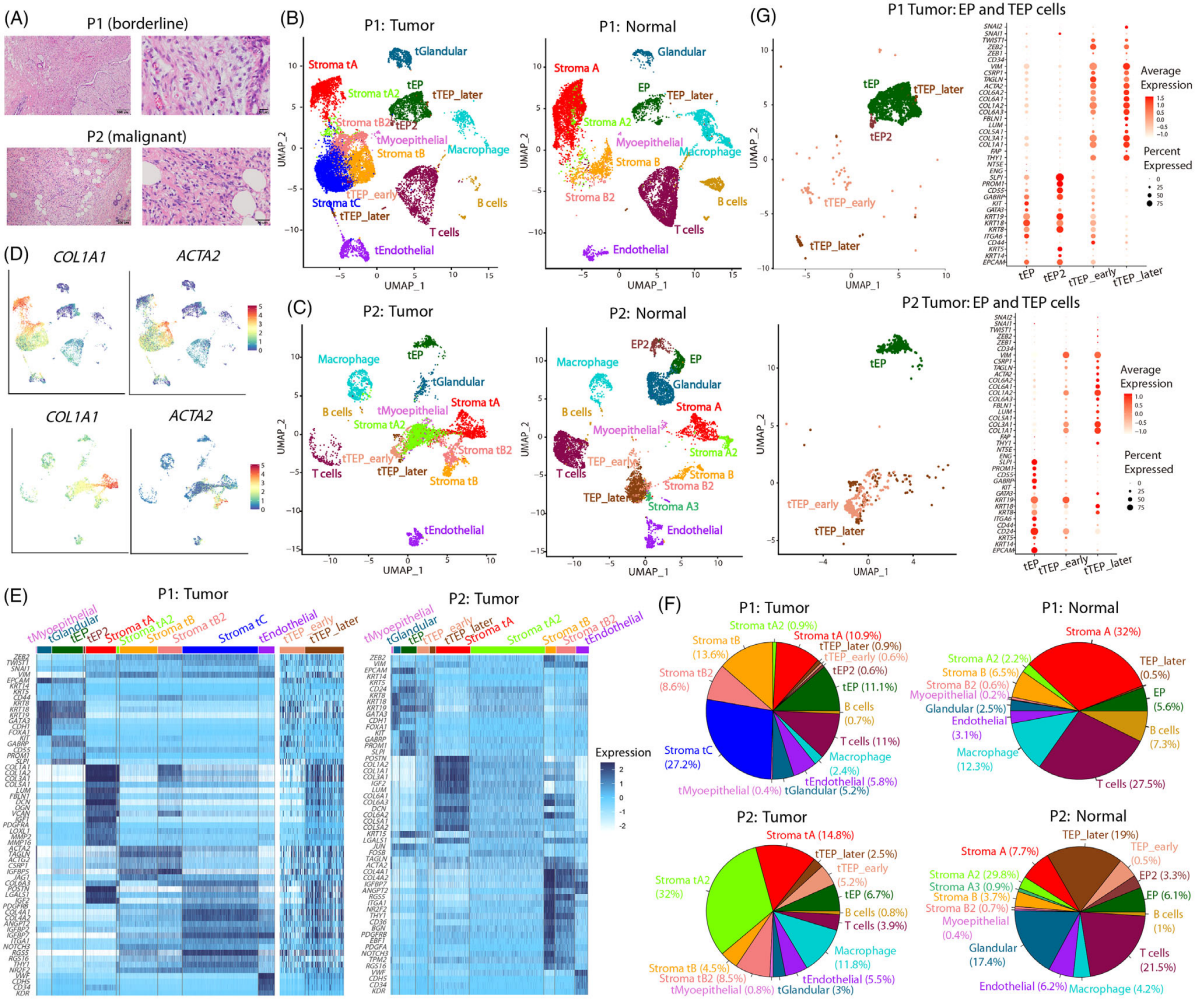
Single‐cell classification. (A) Haematoxylin and eosin (H&E) staining of tumour tissue sections from a patient with borderline phyllodes tumour (PT) (P1) and a patient with malignant PT (P2). (B and C) Uniform manifold approximation and projection (UMAP) plots of cells from tumour and normal tissues in P1 (B) and P2 (C). Except for immune cells, we added the character ‘t’ to the cell cluster name to indicate the cells in the tumour tissue. (D) Expression levels of *COL1A1* and *ACTA2* across cells illustrated in UMAP plots from tumour tissues of P1 (top) and P2 (bottom). (E) Heatmap of marker gene and signature gene expression in epithelial, stromal and endothelial cells. In particular, Stroma tA cells expressed signatures of cancer‐associated fibroblast (CAF) such as *PDGFRA*, *MMP2*, *MMP16* and *LOXL1*, and insulin‐like growth (IGF) factors such as *IGF1* and *IGF2*. Stroma tB expressed *CSRP1*, *IGFBP5* and *JAG1*, and Stroma tC highly expressed *PDGFRB*, *IGF2*, *COL4A1/2* and *IGFBP7*. (F) Proportion of all cell populations in each sample. (G) UMAP plots of epithelial progenitor (EP) and transitioning EP (TEP) cells from tumour tissues and dot plots of marker gene expression in these cells.

To decipher the intratumoural heterogeneity in breast PTs, we collected fresh primary tumours and adjacent normal tissues for scRNA‐seq using the 10× Genomics Chromium platform (Figure 1). Patient diagnoses were assessed using strict histological criteria (Figure 2A). In total, 52 025 single cells were isolated and sequenced with a median depth of 38 931 reads per cell, with 35 431 cells passing quality control. The main cell types in the tumour and adjacent normal tissues were similar and included myoepithelial cells, glandular cells, epithelial progenitors (EPs), stromal cells, endothelial cells and immune cells (Figure 2B,C). Gene Ontology (GO) annotation showed that stromal cell function was mainly enriched in the organisation of the extracellular matrix (ECM) (Figure S1). In addition, immune cells include T cells, B cells and macrophages (Figure S2A).

Importantly, the composition of stromal cell subpopulations showed great differences between tumour and normal tissues. Two major stromal subpopulations were observed in normal tissues, including Stroma A expressing known representative fibroblast markers, such as *COL1A1*, and Stroma B marked with myofibroblast marker *ACTA2* (Figures 2B,C and S2B). They were recapitulated in tumour tissues and named Stroma tA and Stroma tB (‘t’ was added to indicate cells in tumour tissue), respectively (Figure 2B–D). Two additional stromal subpopulations, Stroma tA2 and Stroma tB2, were clustered close to and shared signature genes with Stroma tA and Stroma tB cells, respectively (Figure 2B–E). Their cell proportions increased from borderline adjacent normal tissue (2.8%) to borderline tumour tissue (9.5%), malignant adjacent normal tissue (30.5%) and malignant tumour tissue (40.5%) (Figure 2F). Notably, tumour stromal cells in P1 yielded a third major subpopulation, Stroma tC cells, with high expression of the stromal marker *COL6A3* (Figure 2B,E). The CNV analysis based on scRNA‐seq showed evident CNVs in tumour stromal subpopulations, demonstrating the tumourous nature of stromal cells in tumour tissues (Figure S2C).

Unique cells were found clustered close to stromal cells but exhibiting characteristics similar to transitioning EPs (TEP) undergoing EMT: expressing *VIM* and stromal marker genes, including *COL1A1/2*, and marker genes with EPs, including *KRT8*, *KRT18*, *KRT19*, *KIT*, *CD24* and *CD44* (Figures 2G and S2D). Based on the relative expression levels of epithelial and mesenchymal marker genes, we divided them into two subpopulations: tTEP_early and tTEP_later (TEP_early and TEP_later in normal), with the former highly expressing the signature EP genes, and the latter highly expressing the signature genes in stromal cells (Figure 2E). While only a small fraction of TEP cells was found in P1, their proportions in P2 were pronounced, especially in adjacent normal tissues (Figure 2B,C,F).

Except for Stroma tA cells, the remaining stromal subpopulations expressed high levels of *ZEB2*, a marker of EMT (Figure 2E). Additionally, we identified signature genes in tumour stromal cells, including signatures of CAF and insulin‐like growth factors in Stroma tA. Stroma tB in P2 shared signature genes with Stroma tC, including *RGS5*, *ITGA1*, *NR2F2*, *COL4A1/2*, *THY1*, *IGFBP7*, *PDGFRB*, *NOTCH3* and *ANGPT2*. A recent study demonstrated that the PI3K–Akt and MAPK pathways were upregulated in malignant breast PTs^32^; here, our scRNA‐seq data showed that these pathways displayed heterogeneous enrichment in stromal cells (Figure S3).

### 3.2 Stromal subpopulations display distinct differentiation trajectory

**FIGURE 3:**
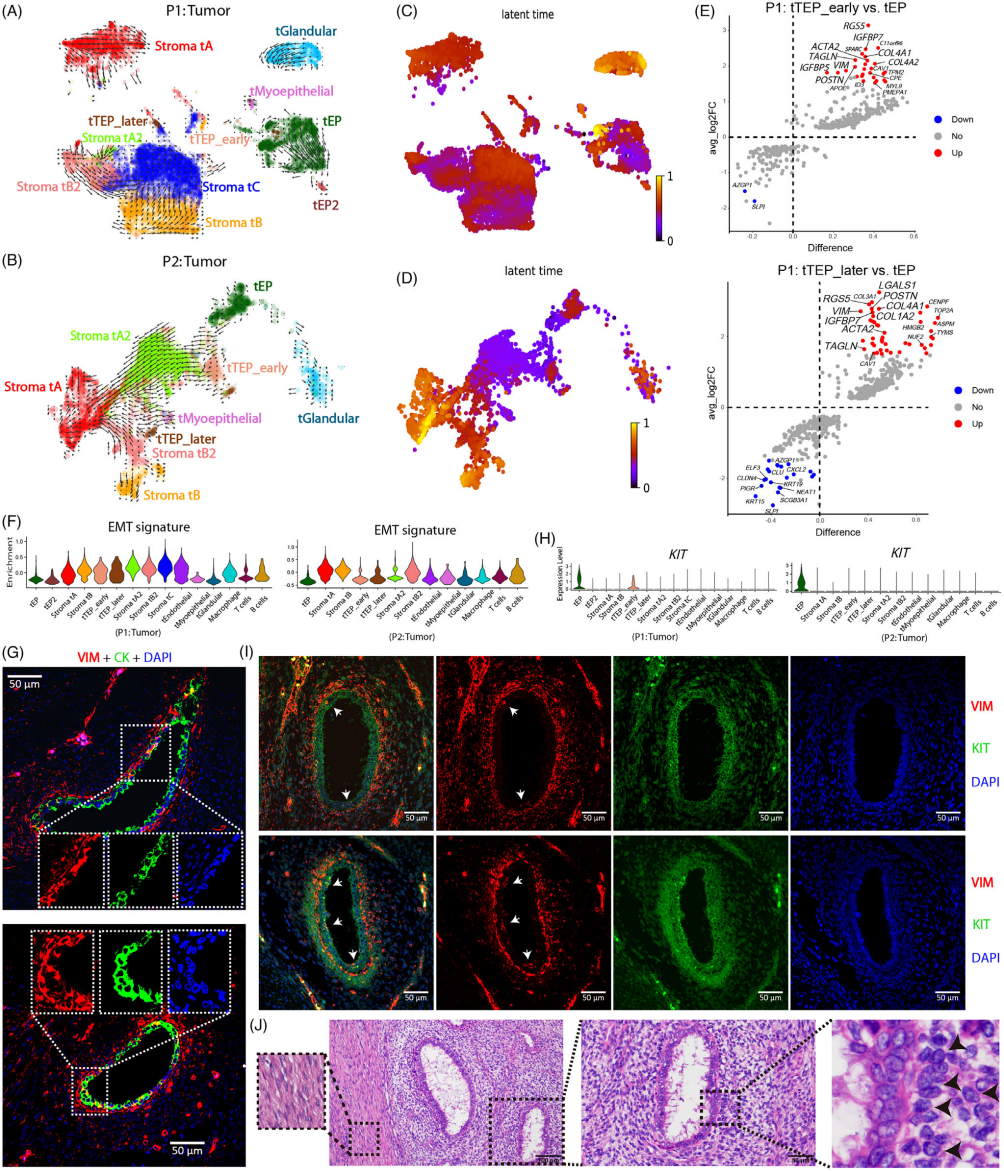
Trajectory of stromal and epithelial cell lineage differentiation in tumour tissues. RNA velocity of stromal and epithelial cells in P1 (A) and P2 (B) tumour tissues. The arrows represent the direction of transcriptional differentiation. Latent times of differentiation are shown in (C) and (D) accordingly. (E) Differential gene expression analysis between transitioning epithelial progenitor (TEP) cells and epithelial progenitors (EPs) in P1 tumour tissue. The *x*‐axis shows the difference in the proportion of cells where the gene is detected between the two groups. The *y*‐axis shows the log fold‐change of the average expression between the two groups. (F) Distribution of epithelial–mesenchymal transition (EMT) signature gene enrichment scores in cell populations. (G) Immunofluorescence (IF) staining of VIM and CK in tumour tissue from a malignant breast phyllodes tumour (PT). (H) Expression of *KIT* in cells. (I) IF staining of VIM and KIT. (J) Haematoxylin and eosin (H&E) staining of the region in (I).

To explore the lineage relationship between tumour cells, we inferred a transcriptional trajectory describing gene expression change direction in each cell for each patient. Early on, EPs switched their transcriptional state, and the transcriptional changes of tTEP_early occurred earlier than those of tTEP_later cells (Figure 3A–D). EPs in P2 were found to differentiate into tTEP_early cells (Figure 3B). Following the onset of TEP cells was their differentiation into Stroma tA2 and Stroma tB2 cells. In P1, evident differentiation of Stroma tA2 into Stroma tB2 cells was observed, followed by Stroma tB2 into Stroma tC cells (Figure 3A). Stromal tB cells eventually differentiated into Stroma tC cells through an alternative differentiation path. In contrast, Stroma tA did not appear to have a direct differentiation relationship with these stromal cells. In P2, we observed transcriptional changes in Stroma tA2 towards Stroma tA cells (Figure 3B), suggesting that their differentiation may be a source of Stroma tA cells. In addition, Stroma tB differentiated in a different direction, the transcription of which may eventually differentiate towards that of Stroma tC cells, given that they shared many of the same signature genes. In adjacent normal tissues, the earliest cells were EPs and TEP cells (Figure S4A–D), and we found that EPs harboured two subpopulations with a sequential order in the latency of their transcriptional changes (Figure S4B,D). Similar to those in tumour tissues, except for Stroma A and Stroma B cells, differentiation relationships existed between other stromal cells, and between them and TEP cells.

Signature genes of Stroma tC, including *RGS5*, *COL4A1/2*, *IGFBP7*, *NR2F2* and *VIM*, were upregulated in TEP cells compared to EPs (Figures 3E and S4E–F). The EMT scoring of cells showed a gradual increase from tTEP_early to the final differentiated Stroma tC cells (Figure 3F). Notably, the relatively high EMT scores of Stroma tA cells in P2 may be due to the mixing of differentiated stromal cells. Furthermore, IF staining showed co‐expression of cytokeratin (CK) and vimentin (VIM) in cells located on the side of some glands near the stroma (Figure 3G), demonstrating the existence of EMT in these cells. As *KIT* was particularly highly expressed in EPs and expressed in tTEP_early cells (Figure 3H), we detected KIT and VIM through IF staining in the same region as shown in Figure 3G. Consistently, EPs in the glands were observed to undergo a mesenchymal transition as they expressed *VIM* (Figure 3I). We also observed co‐localisation of *KIT* and *VIM* expression in stromal cells located in the zone adjacent to the epithelium (ZAE), verifying the presence of TEP cells. They are not typically spindle shaped but show an ovoid epithelial cell morphology (Figure 3J).

In summary, these findings suggest that both EPs and stromal cells underwent prominent transcriptional changes in breast PTs. A clear transcriptional differentiation relationship was noted between the stromal subpopulations, while EPs exhibited mesenchymal transition.

### 3.3 Differentiation and intercellular cooperation are mediated by growth factors and collagen

**FIGURE 4:**
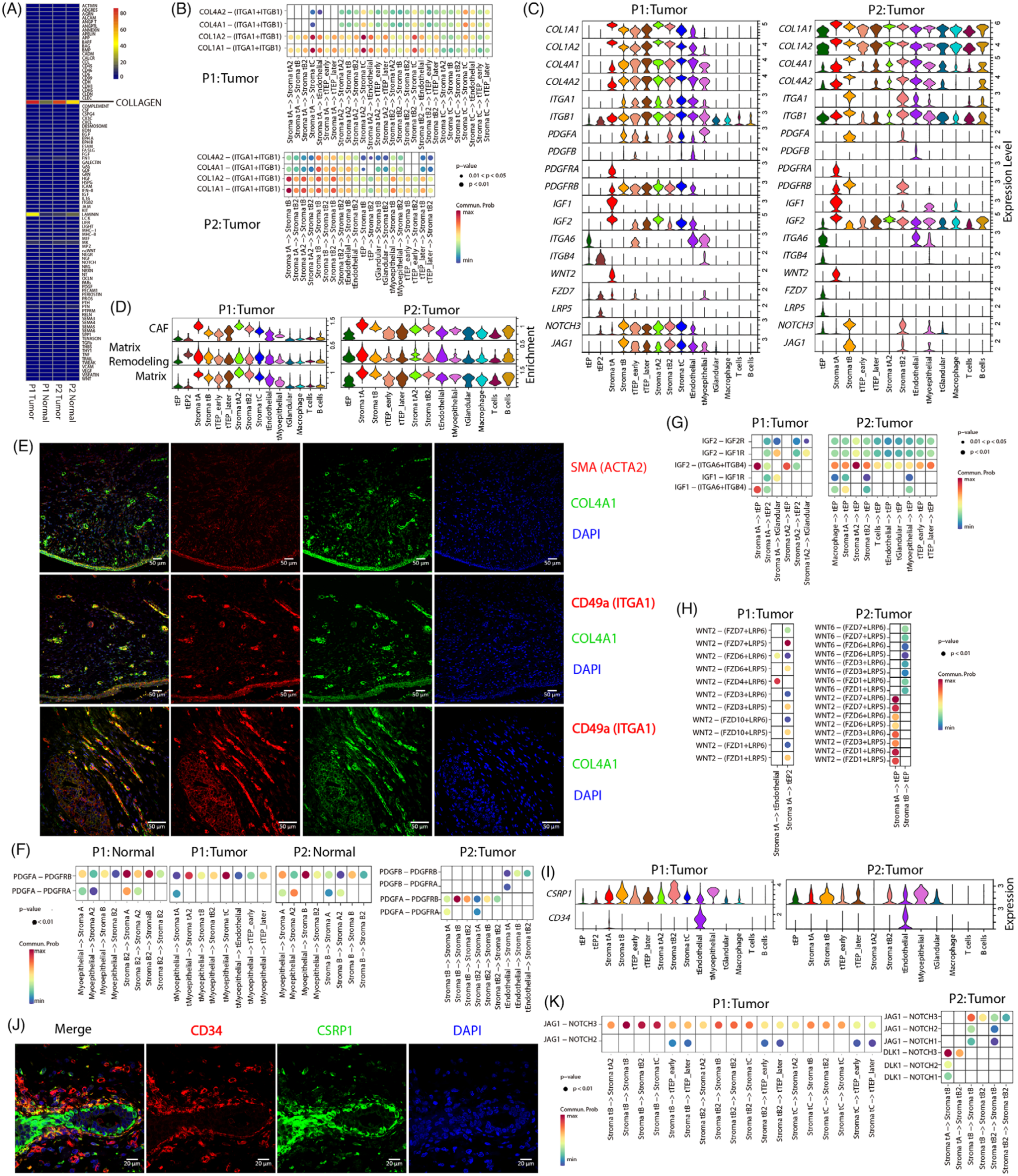
Intercellular communication in tumour tissues. (A) Heatmap showing the distribution of intercellular communication probabilities for all pathways in each sample. In each sample, we calculated the sum of communication probabilities between cells for each pathway separately. The rows in the heatmap show the names of the pathways. (B) Ligand–receptor pairs between cells in the COLLAGEN pathway, and the probability and significance of their communication. (C) Violin distribution of expression levels of genes encoding receptors and ligands in each patient's tumour sample. (D) Enrichment scores of signature genes of cancer‐associated fibroblast (CAF), matrix remodelling and matrix in different cells. (E) Immunofluorescence (IF) staining of COL4A1, ITGA1 and ACTA2 in P2 tumour tissue. (F–H) Ligand–receptor pairs between cells in PDGF (F), insulin‐like growth (IGF) (G) and Wnt (H) pathways. (I) Expression of *CSRP1* and *CD34* in different cells. (J) IF staining of CSRP1 and CD34 in P1 tumour tissue. (K) Ligand–receptor pairs between cells in the NOTCH pathway.

We then explored how the cell subpopulations interacted with each other, inferring the probability of mutual communication between cells based on the known ligand–receptor pairs. Among all ligand–receptor pathways, the COLLAGEN pathway had the highest probability of intercellular communication in tumour tissues (Figure 4A). Notably, strong *COL1A1/2*‐mediated interactions were observed between Stroma tA and other cells, whereas progressively stronger *COL4A1/2*‐mediated interactions were observed between differentiated stromal cells along the early and late order of the differentiation (Figure 4B). The expression levels of *COL4A1/2* gradually increased during differentiation; in contrast, the expression of *COL1A1/2* did not increase persistently (Figure 4C). Matrix scoring changed according to the expression levels of *COL1A1/2* during cell differentiation (Figure 4D), suggesting that ECM regulation was mainly dependent on *COL1A1/2*. Both types of collagen bound to the integrins *ITGA1* and *ITGB1*, exhibiting high expression levels in differentiated stromal cells (Figure 4C). IF staining further verified that COL4A1 was highly expressed in ACTA2‐marked Stroma tB and Stroma tB2 cells in P2 and co‐localised with ITGA1 expression (Figures 4C,E and 2E). Furthermore, epithelial cells in glands exhibited co‐expression of *COL4A1* and *ITGA1*, indicating that *COL4A1* was widely expressed in malignant tumour tissues. Thus, the continuous expression of *COL4A1/2* and integrin, along with their binding, accompanied the entire process of stromal differentiation.

Given the expression of platelet‐derived growth factor (PDGF) receptors, insulin‐like binding proteins, and *NOTCH3* in stromal cells (Figure 2E), we carefully examined the cellular communication involving the associated genes. In the adjacent normal tissue, myoepithelial cells communicated with stromal cells through the expression of *PDGFA* to bind *PDGFRA* and *PDGFRB* receptors (Figure 4F). In P1 tumour tissue, myoepithelial cells expressed large amounts of *PDGFA* when in contact with stromal cells (Figure 4C,F). Stroma tA cells express both *PDGFRA* and *PDGFRB* receptors. However, for Stroma tB and those differentiated stromal cells, only *PDGFRB* was expressed, with its expression increasing as differentiation proceeded. Differentiated stromal cells also expressed *PDGFA*, whereas in P2 tumour, *PDGFA* was no longer expressed in myoepithelial cells but was entirely expressed in Stroma tB and Stroma tB2 cells. We also observed that the endothelial cells expressed *PDGFB*. These results demonstrate that the demand for PDGF during differentiation gradually became less dependent on myoepithelial cells; instead, differentiated stromal cells displayed an autocrine response to meet the supply of PDGF. The growth of Stroma tA cells initially benefited from *PDGFA* expression by myoepithelial cells and then depended on other stromal cells.

Intensive communication was observed between Stroma tA and EPs, where Stroma tA expressed high levels of *IGF1* and *IGF2*, and their receptors *ITGA6* and *ITGB4* were abundantly expressed by EPs (Figure 4C,G). Differentiated stromal cells persistently expressed high levels of *IGF2* (Figure 4C), which further bound to *ITGA6* and *ITGB4* receptors on EPs. Our trajectory analysis showed that tEP2 differentiated from tEP cells in P1 (Figure 3A); here, tEP2 expressed higher *ITGB4* than did tEP cells (Figure 4C), which facilitated binding with *IGF2*. Additionally, the communication between Stroma tA and EPs was mediated by *WNT2*, and specifically in P1, *WNT2* targeted tEP2 cells (Figure 4H).

We noted that in addition to endothelial cells, *CD34* was expressed in Stroma tA cells in P1 (Figure 4I). IF staining for CD34 showed that Stroma tA cells were close to the gland (Figure 4J). Stroma tA cells were highly enriched in CAF signature genes (Figure 4D). The acquisition of CAF properties may be attributed to the continuous stimulation of PDGF. Consistent with the differentiation of Stroma tA2 into Stroma tA cells, Stroma tA2 cells also exhibited high CAF scores (Figure 4D). Furthermore, the differentiated stromal cells were in contact with each other through strong *JAG1* and *NOTCH3* interactions (Figure 4C,K). In addition, TEP and differentiated stromal cells may evade potential immune cell surveillance by reducing or even losing *MDK* ligands (Figure S5).

These results demonstrate that Stroma tA cells and EPs have strong communication, whereas differentiated stromal cells maintain intercellular interactions with each other mainly through the expression of *COL4A1/2* and growth factors.

### 3.4 ST data show spatial growth pattern of differentiated stromal cells in tissue

**FIGURE 5:**
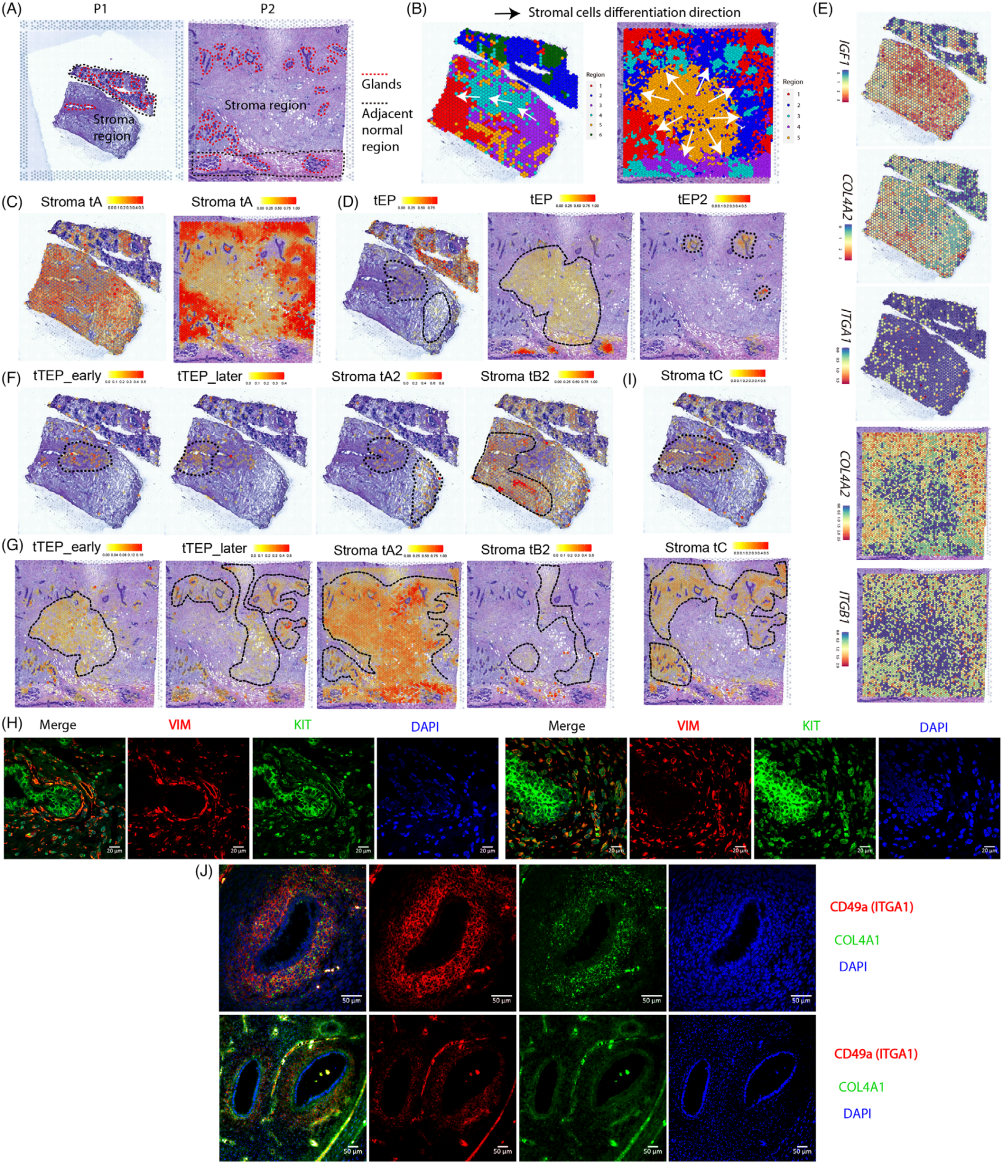
Spatial mapping of cell populations based on spatial transcriptome (ST) data. (A) Haematoxylin and eosin (H&E) staining of tissue sections of ST spots in P1 and P2. The dashed lines mark glands (red) and normal tissues (black). (B) Region classification of the ST data for P1 (left) and P2 (right). We obtained 1465 and 4955 spots under the tissue and detected a median depth of 6806 and 3632 unique molecular identifiers per spot, and 2731 and 1448 genes per spot for P1 and P2, respectively. Spots were classified into different regions using the negative matrix factorisation (NMF) method. Each region is marked with a different colour. We illustrated the direction of stromal cell differentiation on tissues with arrows based on stromal cell differentiation relationships as well as their spatial distribution in tissues (D, F, G). (C) Spatial distribution of Stroma tA cells of each of the two patients based on their ST data. (D) Distribution of epithelial progenitors (EPs) based on ST data of two patients. The dashed line marks their distribution area in tumour tissue. (E) Expression distribution of *IGF1*, *COL4A2*, *ITGA1* and *ITGB1* in ST data. (F and G) Spatial distribution of transitioning EP (TEP) (tTEP_early and tTEP_later) and differentiated stromal cells (Stroma tA2, Stroma tB2 and Stroma tC) of each of the two patients in their ST data. (H) Immunofluorescence (IF) staining of VIM and KIT in tumour tissues from P1 (top) and P2 (bottom). (I) Distribution of Stroma tC cells on ST data of two patients. (J) IF staining of ITGA1 and COL4A1 in the same tumour region in Figure 3I from a malignant breast phyllodes tumour (PT).

To further characterise the spatial organisation of the cells, we analysed the ST with sections of freshly frozen tumour tissues together with a few areas of adjacent normal tissues from the same two patients using the 10× Genomics Visium platform (Figures 1, 5 and S6). We classified the spots into distinct regions (Figures 5 and S7B), and deconvolved the spatial locations of scRNA‐seq cells in ST data for each patient, both of which consistently captured the most basic tissue structures. GO function enrichment for the signatures of each region confirmed the rationality of the cell‐type deconvolution (Figure S8).

The ST of P2 represented a larger tissue area, whereas the ST of P1 described more details about the gland and stroma. Stroma tA cells were widely distributed in both tumour tissues (Figure 5C), which was consistent with the enrichment of CAF signature genes in ST (Figure S7C). EPs were located in some normal glands, but some were found in the tumour stroma (Figure 5D). Given the transcription change of tEP into tEP2 (Figure 3A), EPs grew from the stroma in the direction of ZAE. The expression of *IGF1*, the ligand of Stroma tA, overlapped with that of the EP receptors (Figures 5E and S7D), verifying a spatial interaction between them. The distribution of tTEP_early cells overlapped with that of EPs in the tumour tissue, and tTEP_later cells showed the same growth pattern in the direction of ZAE (Figure 5F,G). The distribution of TEP cells in ZAE was consistent with that shown in Figure 3I. IF staining of KIT and VIM showed that TEP cells were also present in the tumour stroma (Figure 5H), confirming the distribution of TEP cells in the ST data. The overlap of the expression of PDGF and its receptors in the ST verified the cell–cell communication via PDGF in differentiated stromal cells (Figure S7D).

Stroma tA2 and Stroma tB2 cells emerged from the regions corresponding to where tTEP_early and tTEP_later are located (Figure 5F,G). They infiltrated in the direction of the surrounding glands and even into adjacent normal tissues. Stroma tC cells formed a distinct distribution in ZAE (Figure 5I). Thus, the spatial distribution of stromal cell subpopulations and their growth patterns agreed with their differentiation trajectory. IF further demonstrated the distribution of stromal cells adjacent to the glands (Figure S9A). The expression of *COL4A2* increased in the same direction as the spatial differentiation of the stromal cells and overlapped with its receptor *ITGA1* (Figure 5E,B). Furthermore, IF staining of COL4A1 and ITGA1 in tumour tissue sections from the same region (Figure 3I) revealed a unique structure of stromal cells surrounding the glands (Figure 5J). In terms of their expression in cells (Figure 4C), many ITGA1 and COL4A1 formed a meshwork outside the glandular cells, inside which many stromal cells were arranged with high expressed levels of both proteins. Therefore, the anchoring of type IV collagen and integrins provides a structural channel for the migration of stromal cells towards the glands. In addition, since *COL4A1/2* is commonly expressed in the basement membrane (BM), we noticed that COL4A1 did not mark a complete BM as in glands of benign and borderline tumours (Figure S9B).

Integrating spatial localisation within the tissue, our findings reveal a growth pattern of differentiated stromal cells extending from the stroma towards surrounding glands, particularly emphasising the facilitating role of *COL4A1/2* binding with *ITGA1/B1* in this process.

### 3.5 *COL4A1/2* expression accompanies stromal cell differentiation and confers proliferation capacity

**FIGURE 6:**
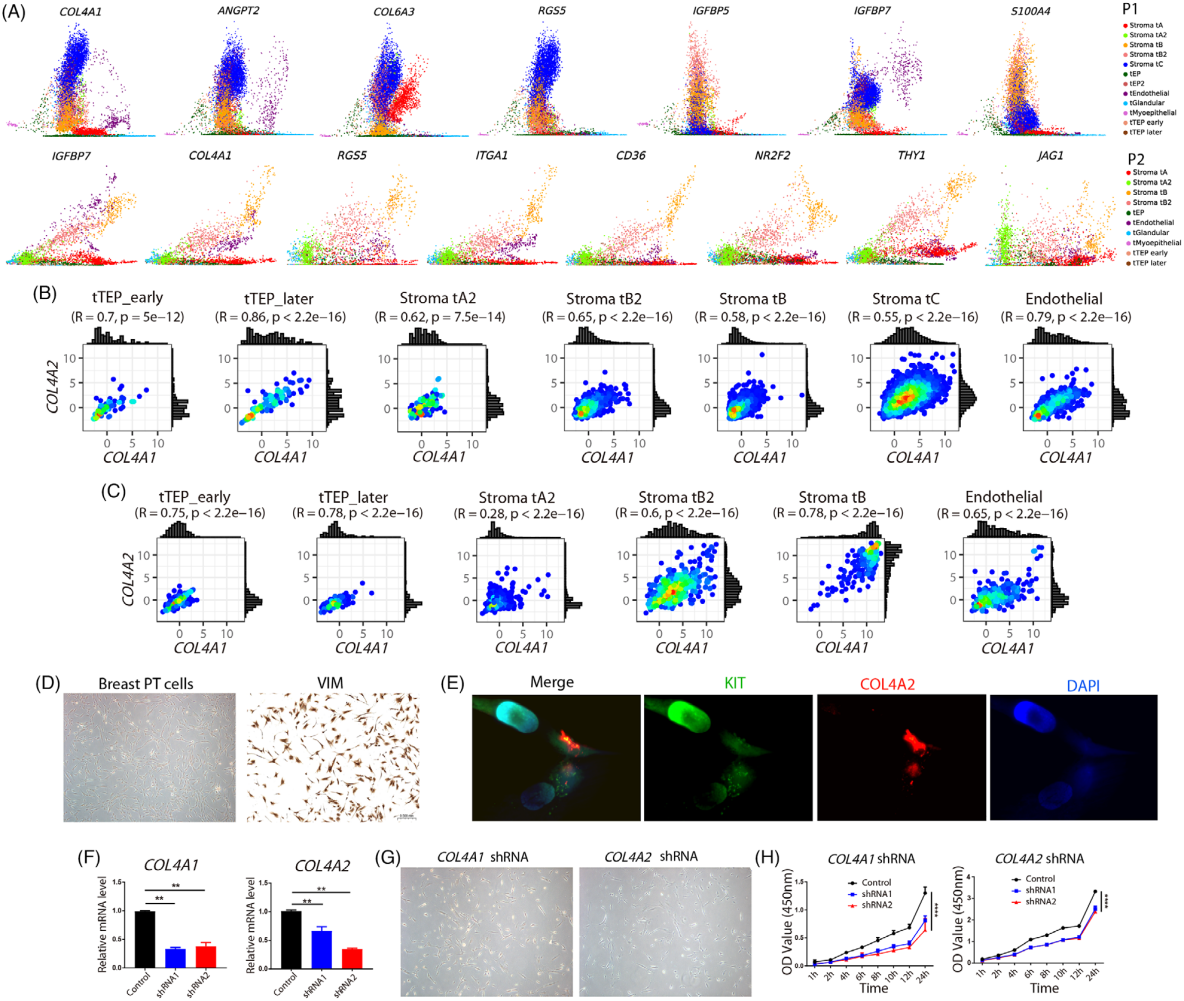
Evaluation of expression levels of *COL4A1/2*. (A) Genes with the most dominant expression change along with latent time in differentiated stromal cells were obtained from scVelo results. Scatter plots showing the expression levels for each gene in different cells along latent time. Each dot represents a cell, and different colour mark different cell subpopulations. (B and C) Distribution of *COL4A1* and *COL4A2* expression in stromal and endothelial cells in P1 (B) and P2 (C). Pearson correlation coefficients and *p*‐values are shown above each scatterplot. (D) Culture of breast phyllodes tumour (PT) cells, and immunohistochemistry (IHC) staining showing they are VIM positive. (E) Immunofluorescence (IF) staining of KIT and COL4A2 in breast PT cells. (F) Inhibition of *COL4A1* and *COL4A2* expression in tumour cells. The shRNA with nonsense sequence was used as a negative control. (G) Growth status of breast PT cells at 3 days after inhibition of *COL4A1* and *COL4A2* expression. (H) *COL4A1* and *COL4A2* expression inhibition reduced the proliferative activity of tumour cells. Tukey's multiple comparisons test was performed to compare differences between groups. ^**^ *p* < .01, ^***^ *p* < .001, ^****^ *p* < .0001.

We then investigated the function of *COL4A1/2* in stromal cell differentiation and proliferation. We first aimed to identify transcriptional genes that define cellular differentiation. Based on the scRNA‐seq data, we identified genes exhibiting dominant expression changes in different cells with latent time (Figure 6A). The expression of *COL4A1*, *IGFBP7* and *RGS5* governed the progression of differentiated stromal cells. In tandem with stromal cell differentiation, we carefully examined the expression distribution of *COL4A1* and *COL4A2* (Figure 6B,C). Their expression levels were well correlated and increased synchronously with high correlation coefficients in TEP cells. Their distribution shifted from right‐skewed to normal from Stroma tA2 and Stroma tB2 to Stroma tC. In P2, their expression levels increased persistently and exhibited a left‐skewed distribution in Stroma tB cells. By examining the correlation between the expression of other genes with dominant expression changes and *COL4A1*, we found that their expression increased lagging behind that of *COL4A1/2* (Figures S10 and S11; Supporting Information). Consequently, the increase in *COL4A1/2* expression may be the earliest transcriptional change that occurred during stromal cell differentiation and remained highly expressed along with differentiation.

To further validate the influence of *COL4A1/2* on the stromal cell proliferation, we prepared primary tumour cell culture using fresh tumour tissues from patients (Figures 1 and 6D). These cells were confirmed to be positive for KIT and COL4A2 staining, validating the presence of TEP cells in in vitro culture and their expression of *COL4A2* (Figure 6E). Using shRNA fragments, we inhibited the expression of *COL4A1* and *COL4A2* (Figure 6F). Subsequent cell proliferation assays verified that reduced expression of these two genes led to decreased cell proliferation (Figure 6G,H).

### 3.6 Active transcription in differentiated stromal cells

**FIGURE 7:**
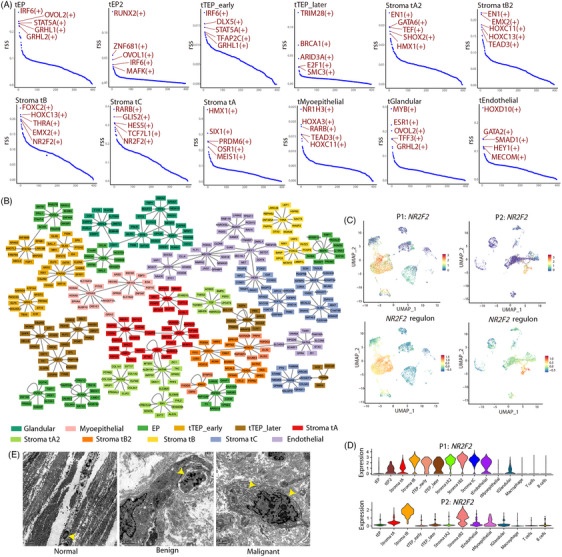
Transcriptional regulatory relationship in P1. (A) Top five transcription factors (TFs) specific to epithelial, stromal and endothelial cells in P1. (B) TF and target gene regulatory network among epithelial, stromal and endothelial cells in P1. Different colour mark different cell populations. (C) Uniform manifold approximation and projection (UMAP) plots of *NR2F2* expression level and its regulon activity in tumour tissues. (D) Violin plots of *NR2F2* expression in different cell populations. (E) Transmission electron microscopy (TEM) images of fibroblast structures in normal, benign and malignant breast phyllodes tumour (PT) tissue. The arrows indicate the nucleus and endoplasmic reticulum.

We then investigated the underlying transcriptional regulatory relationships between stromal subpopulations. Using the top five cell population‐specific transcription factors (TFs) and the top 10 target genes for each TF, we constructed regulatory networks between different cell populations. Overall, EPs shared several TFs with TEP cells, particularly tTEP_early (Figures 7A and S12A), suggesting a similarity in transcriptional regulation between them. *E2F1* and *BRCA1* were found to encode tTEP_later cell‐specific TFs with close regulatory relationships (Figures 7B and S12B). The gene that encodes the nuclear receptor TF, *NR2F2*, was specific to both Stroma tB and Stroma tC cells (Figure 7A), with high expression levels and high regulon activity (Figure 7C). This gene was highly expressed in the differentiated stromal cells (Figures 2E and 7D). Additionally, we noticed that *NR2F2* was highly expressed in tEP2 cells compared with tEP cells (Figure 7D), suggesting its association with transcriptional changes in EPs. Its target genes, *PDGFRB* and *IGFBP5* (Figure 7B), were active in differentiated stromal cells in P1, as well as in Stroma tB cells of P2 (Figure 2E). TEM revealed that in contrast to the normal nuclei structure of fibroblasts in normal breast and benign PT tissues, malignant fibroblasts exhibited enlarged nuclei with increased nucleoli, expanded rough endoplasmic reticulum, and a large number of ribosomes (Figure 7E). In addition, stromal cells had tight regulatory relationships with endothelial cells, and microvessels were found near the glands (Figures 7B and S12; Supporting Information). In summary, these results suggest high nuclear transcriptional activity in differentiated stromal cells, with concurrent involvement of endothelial cells in the transcriptional regulation of these highly active stromal cells.

### 3.7 Validation of heterogeneity in many samples and assessment of the potential diagnostic value of *COL4A1/2*

**FIGURE 8:**
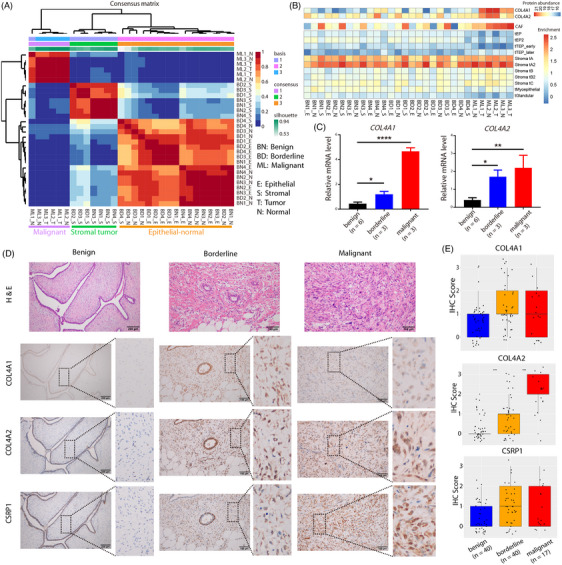
Large patient samples to validate intratumoural heterogeneity and to assess diagnostic value. (A) Clustering of laser capture microdissection (LCM)‐based microproteomic data (Figure 1). BD: borderline; BN: benign; E: tumour epithelial component; ML: malignant; N: adjacent normal tissue; S: tumour stromal component; T: tumour tissue. The number before the underline represents the patient serial number. Samples were classified into three groups: epithelial‐normal (yellow), stromal tumour (green) and malignant (purple) groups. (B) Enrichment of signature genes of cell populations of P1 in the proteomic data. The top row is the enrichment distribution of cancer‐associated fibroblast (CAF) signature genes. (C) Distribution of RT‐qPCR expression values of *COL4A1* and *COL4A2* in benign, borderline and malignant tumour tissues, respectively. Tukey's multiple comparisons test was performed to compare the differences between groups. ^*^ *p* < .05, ^**^ *p* < .01, ^***^ *p* < .001, ^****^ *p* < .0001. (D) Haematoxylin and eosin (H&E) staining and representative immunohistochemistry (IHC) staining of COL4A1, COL4A2 and CSRP1 in benign, borderline and malignant breast phyllodes tumour (PT) tissues. (E) Box plot with jitter illustrating the IHC scores of each protein at different grades. Each dot represents the corresponding IHC score in a patient.

To understand whether intratumoural heterogeneity reflected inter‐patient heterogeneity in breast PTs, we collected tumour and adjacent normal tissues from an additional independent population of four benign, four borderline and three malignant patients (Figure 1). For benign and borderline patients, the LCM technique was used to separate the epithelial and stromal components from the tumour tissue. Microproteomic data of each sample were obtained using DDA mass spectrometry (Figure S13A), and they were classified into three major groups: epithelial‐normal, stromal tumour and malignant groups (Figure 8A). Benign and borderline stromal components exhibited similar protein abundance and were classified into one group. In the epithelial‐normal group, we noticed that subtle protein changes persisted between the epithelial components and adjacent normal tissues in borderline tumour tissues, indicating transcriptional changes in adjacent tissues in borderline tumours. In contrast, the adjacent normal tissues of malignant patients exhibited protein abundance closer to that of the tumour tissue. This observation is consistent with our finding that TEP cells were present in adjacent normal tissues. Importantly, the signature genes of differentiated stromal cells were highly enriched in patients with malignancy, whereas epithelial cell signature genes were weakly or not enriched in patients with malignancy (Figures 8B and S13B), suggesting that stromal cell differentiation contributes to the malignant progression. The signature genes of CAF and Stroma tA cells were also highly enriched in malignant patients.

Signature proteins were extracted from each group (Figure S13C), and the functional enrichment analysis of the signature proteins in the stromal tumour group mainly highlighted processes related to ECM and collagen organisation (Figure S13D). Therefore, alterations in ECM and collagen production were identified as the common major functional changes in stromal differentiation, as well as the generation of inter‐patient heterogeneity in breast PTs. The expression of signature proteins of the stromal tumour group in the single cell and ST data showed that they can reflect stromal cell composition and regional structure (Figure S13E). This proved the rationality of the classification of single‐cell subgroups and spatial regional division, demonstrating that the intratumoural heterogeneity revealed in our scRNA‐seq data exhibited consistent transcriptional changes with inter‐patient heterogeneity.

Finally, given the importance of collagen products, especially *COL4A1/2*, we evaluated their potential diagnostic value in patient grading. Fresh tumour tissues from six benign, three borderline and three malignant cases were analysed via RT‐qPCR (Figure 8C), revealing a positive correlation between *COL4A1/2* expression and patient grade. This observation was consistent with the proteomic data, which indicated high expression in malignant PTs (Figure 8B). Using paraffin tissues from 40 benign, 40 borderline and 17 malignant PT cases, we conducted a large‐scale IHC validation (Figure 8D and Table S1). The analysis confirmed the low expression of *COL4A1/2* in benign tumour tissues and their high expression in borderline and malignant tissues (Table S2; chi‐square test or Fisher's exact test, *p* < .05), especially *COL4A2* (Figure 8E). Therefore, *COL4A1/2* expression was positively correlated with the malignant progression of breast PTs. The expression of *CSRP1*, a signature gene of Stroma tB cells (Figure 2E), displayed the same pattern, supporting their differentiation to promote tumour development. The proteins encoded by these genes displayed similar staining pattern in tumour cells: weakly positive cytoplasmic in benign tissues and intense cytoplasmic and nuclear staining in borderline and malignant tissues. This is consistent with active nuclear transcription in malignant tumour cells. Our data demonstrate that *COL4A1/2* expression accompanies stromal cell differentiation and tumour malignant progression and shows distinct staining patterns in different grades of tumour tissues, thus representing a potential diagnostic marker to distinguish different grades of breast PTs.

## 4 DISCUSSION

**FIGURE 9:**
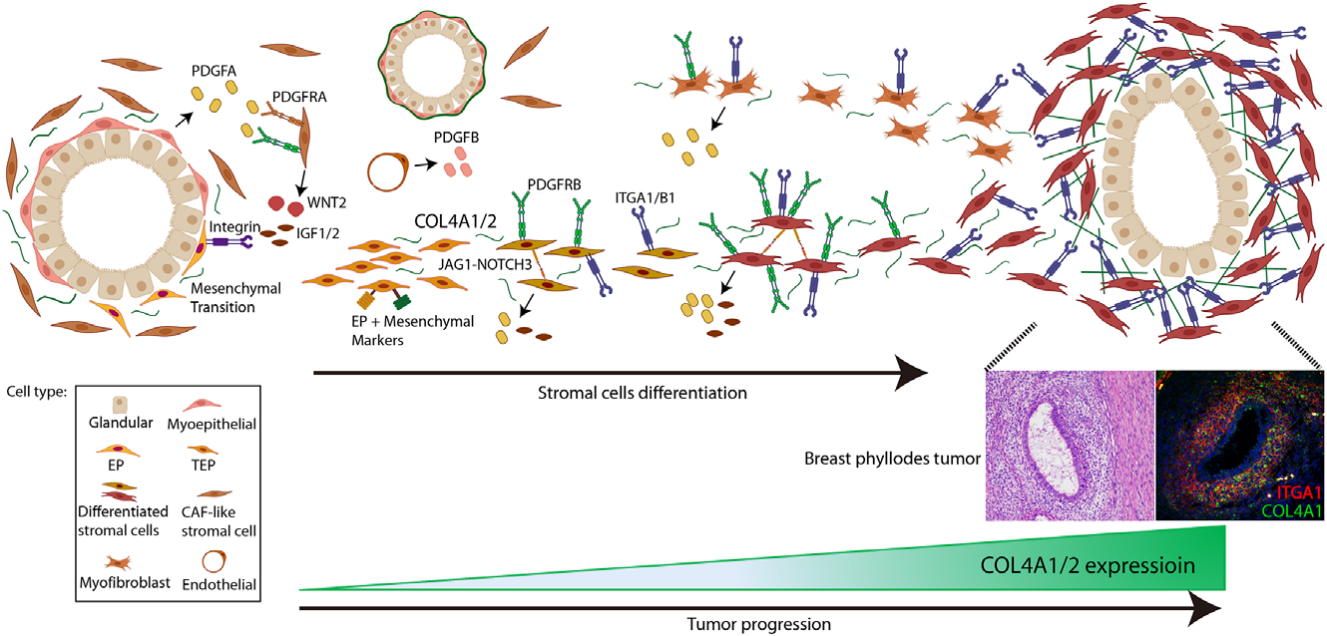
Summary of the pathogenesis of breast phyllodes tumours (PTs). The organisation of breast PTs consists of the epithelial component of the gland and stromal component, in which the epithelial component includes myoepithelial, glandular epithelial and epithelial progenitor (EP) cells, while the stromal cells are distributed in the stromal component. In this study, we found four major transcriptional clusters in tumour stromal cells including cancer‐associated fibroblast (CAF)‐like cells (Stroma tA), myofibroblasts (Stroma tB), transitioning EP (TEP) cells (tTEP_early and tTEP_later) and differentiated stromal cells (Stroma tA2, Stroma tB2 and Stroma tC). The myoepithelial cells interacted with CAF‐like cells via PDGF, which in turn communicated with EPs via WNT2 and insulin‐like growth 1/2 (IGF1/2). EPs were found to have a mesenchymal transition. TEP cells demonstrated a hybrid transcription state, expressing markers for both EPs and mesenchyme. These cells were inferred to further differentiate into transcriptionally active stromal subpopulations continuously expressing *COL4A1/2* and *ITGA1/B1*. In addition, they highly expressed *PDGFA* and *PDGFRB* in an autocrine manner and maintained contact with each other by expressing *NOTCH3*. The binding of *COL4A1/2* with *ITGA1/B1* facilitated a growth pattern of the differentiated stromal cells from the stroma towards surrounding glands that represent the typical morphology of breast PTs. Trajectory analysis inferred that the transcription of myofibroblasts demonstrated a similar differentiation to that of the differentiated stromal cells. Furthermore, large patient samples verified that the protein abundance level of *COL4A1/2* was positively correlated with the grade of breast PTs. Thus, the expression level of *COL4A1/2* can reflect the differentiation status of stromal cells and the degree of malignancy of tumour development, assisting in the accurate diagnosis of breast PTs. Created with BioRender.com.

Unlike breast cancer, breast PTs are tumours of non‐epithelial tissue origin. The molecular basis of their cellular composition and organisation remains unclear. Previous studies on breast PTs have attempted to find molecular evidence of pathogenicity, including genetic mutations,^13^, ^14^, ^15^ epigenetic markers and immunohistochemical indicators^33^; however, none of these studies aimed to link intrinsic heterogeneity formation. Although heterogeneity in breast PTs has been previously reported,^16^, ^17^, ^18^ the studies conducted are limited. Here, we present the cellular composition of breast PTs at the single‐cell level, their lineage and transcriptional relationships, interaction with each other and spatial growth patterns (Figure 9). Overall, we deciphered the cellular and molecular basis behind pathological tissues, identified key cell populations and genes, *COL4A1/2*, in the progression of breast PTs, and revealed how they differentiate and spatially shape pathological tissue phenotypes. We validated *COL4A1/2* as markers for accurate grading. This study furthers our understanding of the tumourigenic mechanism of breast PTs and has clinical implications for accurate diagnosis.

Tissues of breast PTs include both epithelial and stromal components, but the components that contribute to tumour development remain controversial.^34^ Morphological observations of stromal hyperplasia at the periphery of the gland and active mitosis in stromal cells close to the gland led to the assumption that the two components are interdependent^35^; however, clear evidence from an in‐depth study is lacking. We found four major transcriptional clusters in tumour stromal cells including CAF‐like cells, myofibroblasts, TEP cells and differentiated stromal cells (Figure 9). Intercellular communication exists between epithelial and stromal components, and this may lead to the growth of CAF‐like cells and the proliferation of EPs. It is possible that disruption of the BM resulted in the migration of EPs into the stroma. Once exposed to CAF‐like Stroma tA cells, or so‐called ‘reactive stroma’, EPs may have been further stimulated and underwent a mesenchymal transition. Previous studies have reported the expression of stem cell markers in the stroma.^36^, ^37^ In the present study, we observed that TEP cells expressed both EPs and mesenchymal markers, providing evidence of the presence of less differentiated cells in the stroma. Expression of *PDGFRB* by differentiated stromal cells may help them to bind the large amount of *PDGFA* expressed from myoepithelial cells, thus favoring the maintenance of their differentiation. In malignant PTs, however, myoepithelial cells almost no longer expressed *PDGFA*; in contrast, the differentiated stromal cells possessed an autocrine mechanism to continuously express *PDGFA* as well as the corresponding receptors. Thus, the initial tumourigenesis in stromal cells required epithelial cells; however, they were no longer influenced with the progression of differentiation. The proliferation of glandular cells is often observed in benign breast PTs, probably due to active EP differentiation. However, in malignant PTs, glands are rarely observed; additionally, it is unclear whether a persistent EMT contributes to the reduction of epithelial cells or if it is due to a lack of communication between epithelial and stromal cells, eventually leading to gland shrinkage.

The ECM provides a physical scaffold that supports cellular structures and mediates intercellular communication. In a protein study conducted on 15 breast PTs, differentially expressed proteins between grades were mainly enriched in ECM remodelling.^20^ Our results showed that the ECM was remodelled during differentiation, and fibrillar collagen type I, *COL1A1/2*, was highly expressed in Stroma tA cells, whereas non‐fibrillar collagen type IV, *COL4A1/2*, was particularly highly expressed in differentiated stromal cells. In breast PT tissue, stromal cells are frequently observed to be distributed in ZAE. Here we show a more refined structure of this morphological feature: a meshwork structure outside the glandular cells formed by large amounts of ITGA1 and COL4A1, which facilitated stromal cell movement towards the gland by anchoring type IV collagen and integrins. GO enrichment of the signature genes of Stroma tC cells suggested that they might exhibit an amoeba‐like migration strategy (Figure S1). Although Stroma tA cells were also observed to be distributed near some glands, they may be in greater demand for growth factors secreted by myoepithelial cells. In addition, the high and broad expression of *COL4A1/2* in the stroma may provide a convenient channel to carry out a kind of mesenchymal migration,^38^ facilitating the proximity of stromal cells to glands or blood vessels, as well as possible migration of EPs to the stroma. Therefore, changes in the different types of collagen, especially *COL4A1/2*, open the door for cellular differentiation, allowing them to adopt a potentially more efficient migration strategy.

It should be noted that the immune cell recruitment seems to decrease in tumour tissue as we observed the proportion of immune cells in tumour tissue is less than that in adjacent normal tissue. In addition, the interaction between immune cells and stromal cells is actually not as strong as that between stromal cells. Given that alterations in the ECM and collagen production are main functional changes in the differentiated stromal cells, it might be the accumulation of large amounts of collagen products that forms a protective barrier preventing the infiltration of immune cells. However, more samples are needed to be collected to explore the infiltration pattern of immune cell displays between tumour and normal tissues, as well as between different grades of the tumours.

Surgical resection is the main treatment for breast PTs; however, incomplete resection or inherent heterogeneity of the tumour tissue often leads to tumour recurrence and metastasis. Our study sheds light on potentially effective therapeutic strategies for breast PTs, such as the inhibition of *COL4A1/2*, as well as the use of antiangiogenic agents.^39^ The correlation between *COL4A1/2*, especially *COL4A2*, and tumour grade suggests that they may be used as monitoring indicators during treatment. Moreover, a previous study has shown that atypia, mitoses, overgrowth of stromal cells, as well as surgical margins, can be used as factors in nomogram for prediction of recurrence of breast PTs.^11^ Of these four predictors, the first three represent the malignant behaviour of stromal cells, while the surgical margins mark the infiltration of tumour cells into adjacent normal tissues, all reflecting the malignant progression of the tumour. Consistent with this malignant phenotype, in this study, our ST data showed the presence of differentiated stromal cells (Stroma tA2) infiltrating into adjacent normal tissue. This is indicative of signals of tumour cell spread and early seeding. The malignant progression may be recurred in these tissues. Examination of the existence of differentiated stromal cells, or the presence of stromal cell differentiation, in the tissues left after surgical removal, may be a possible means of screening for early lesions of breast PTs as well as predicting recurrence after treatment.

## 5 CONCLUSIONS

In summary, this study highlights the role of *COL4A1/2* on tumour stromal cell differentiation, proliferation and tissue spatial distribution in breast PTs. Its high expression is an important factor in the development of heterogeneity in the tumour tissue. Moreover, *COL4A1/2* expression is associated with tumour malignant progression. These findings imply that *COL4A1/2* expression reflects the malignant behaviour of tumour cells, which can support the accurate grading of breast PTs.

## AUTHOR CONTRIBUTIONS

*Conceptualisation, project administration, resources and funding acquisition*: Mumin Shao. *Methodology*: Xia Li and Mumin Shao. *Software*: Xia Li. *Investigation*: Xia Li and Mumin Shao. *Validation*: Mumin Shao, Xuewen Yu, Jiaxin Bi, Lu Zhang, Xu Jiang and Zhixin Li. *Formal analysis, data curation and visualisation*: Xia Li and Mumin Shao. *Writing—original draft*: Xia Li. *Writing—review and editing*: Xia Li and Mumin Shao. All the authors have read and approved the final version of the manuscript.

## CONFLICT OF INTEREST STATEMENT

The authors declare they have no conflicts of interest.

## ETHICS STATEMENT

This study was approved from the Institutional Review Board of Shenzhen Traditional Chinese Medicine Hospital (ethics number: K2022‐020‐02). Human breast PTs and adjacent normal tissues were obtained with informed consent.

## Supporting information

Supporting Information

## Data Availability

The raw data of scRNA‐seq and ST generated in this study were deposited in Genome Sequence Archive^40^ in National Genomics Data Center, China National Center for Bioinformation/Beijing Institute of Genomics, Chinese Academy of Sciences (GSA‐Human: HRA004345) that are publicly accessible at https://ngdc.cncb.ac.cn/gsa‐human. The mass spectrometry proteomics data have been deposited to the ProteomeXchange Consortium (http://proteomecentral.proteomexchange.org) via the iProX partner repository^41^ with the dataset identifier PXD041431.
