# Supplementary material for: Integrating single‐cell and spatial transcriptomes reveals COL4A1/2 facilitates the spatial organisation of stromal cells differentiation in breast phyllodes tumours
Source: Clin Transl Med. 2024 Mar 14;14(3):e1611. doi: 10.1002/ctm2.1611 (PMC10938066; doi:10.1002/ctm2.1611)
Supplement: Supplementary file 1 — Supporting Information [file CTM2-14-e1611-s001.docx]

**Supplementary material**

**Integrating single-cell and spatial transcriptomes reveals *COL4A1/2* facilitates the spatial organization of stromal cells differentiation in breast phyllodes tumors**

Xia Li^1,2^, Xuewen Yu^1,2^, Jiaxin Bi^1,2^, Xu Jiang^1,2^, Lu Zhang^1,2^, Zhixin Li^3,4^, Mumin Shao^1,2*^

**This file includes:**

**Supplementary figures and figure legends**

**Supplementary tables**

**Supplementary methods**

**Supplementary contents**

**Supplementary figures AND FIGURE legends**

**
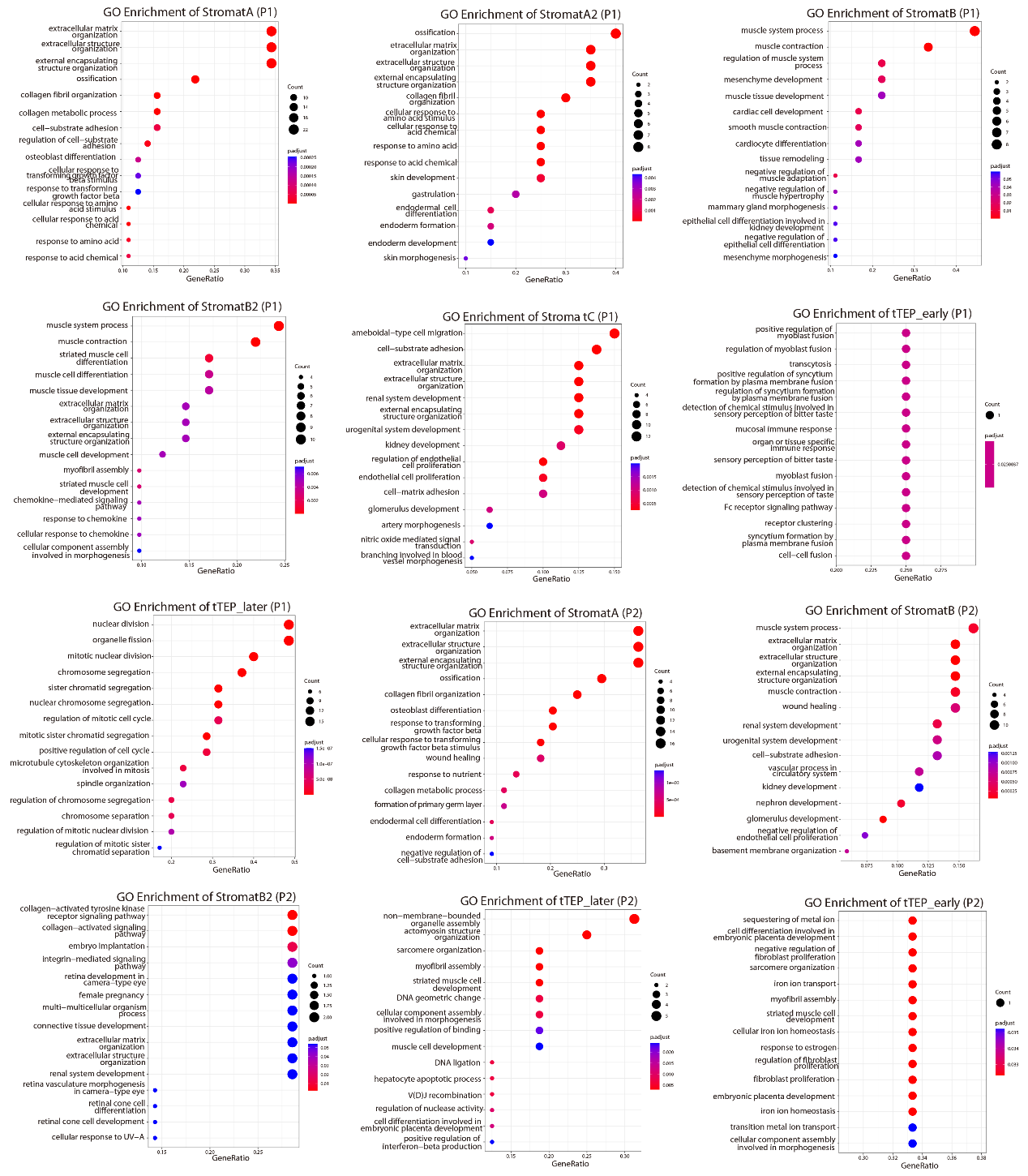
**

**Figure S1. GO functional enrichment of each cell cluster’s signature gene in P1 and P2 tumor tissues.**


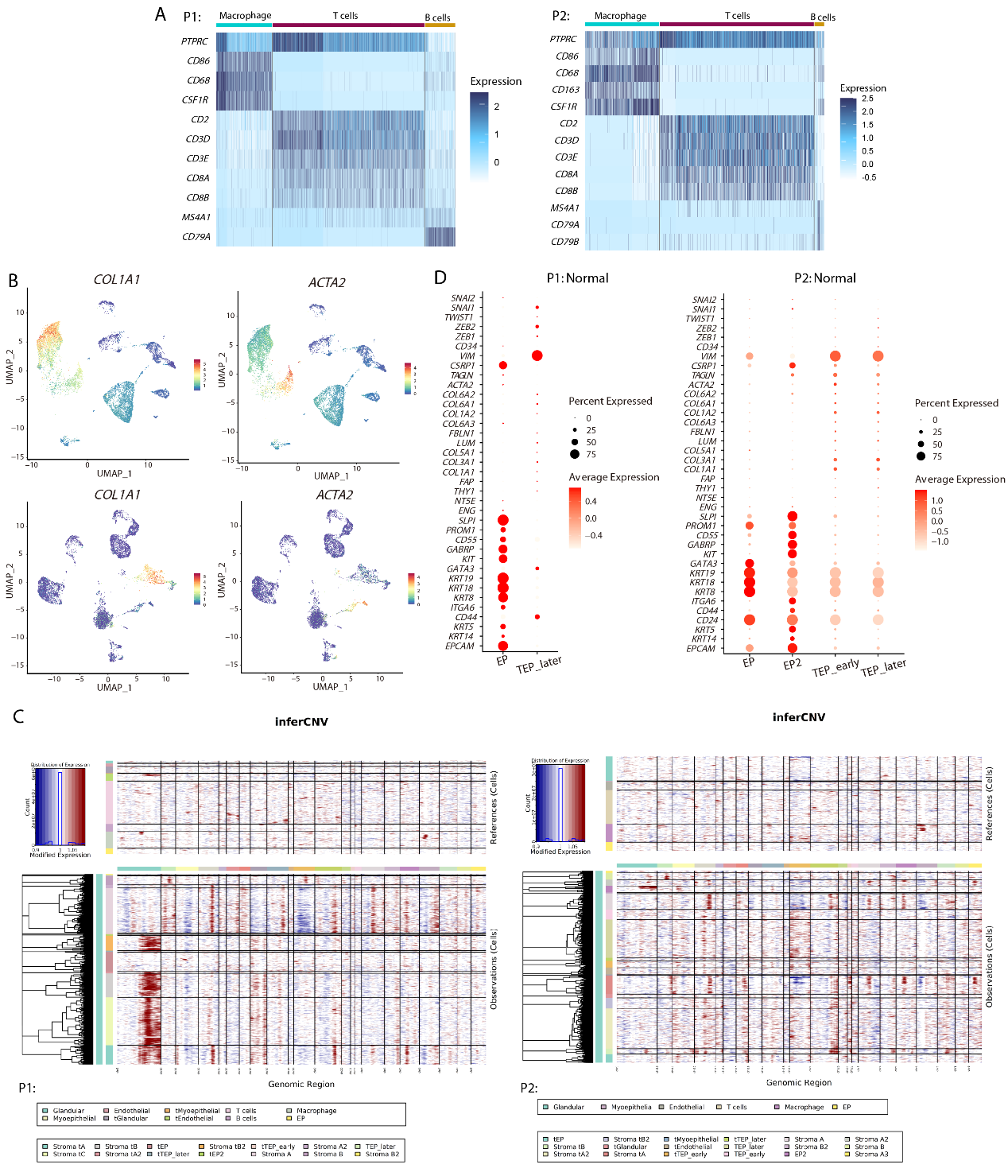


**Figure S2. Marker gene expression assessment and CNV analyses.** (**A**) Heat maps of immune marker gene expression in immune cells. (**B**) UMAP plots of *COL1A1* and *ACTA2* expression levels in adjacent normal tissues of P1 (top) and P2 (bottom). (**C**) CNV distribution maps for each cell type. (**D**) Dot plots of marker gene expression in EP and TEP cells in adjacent normal tissues.


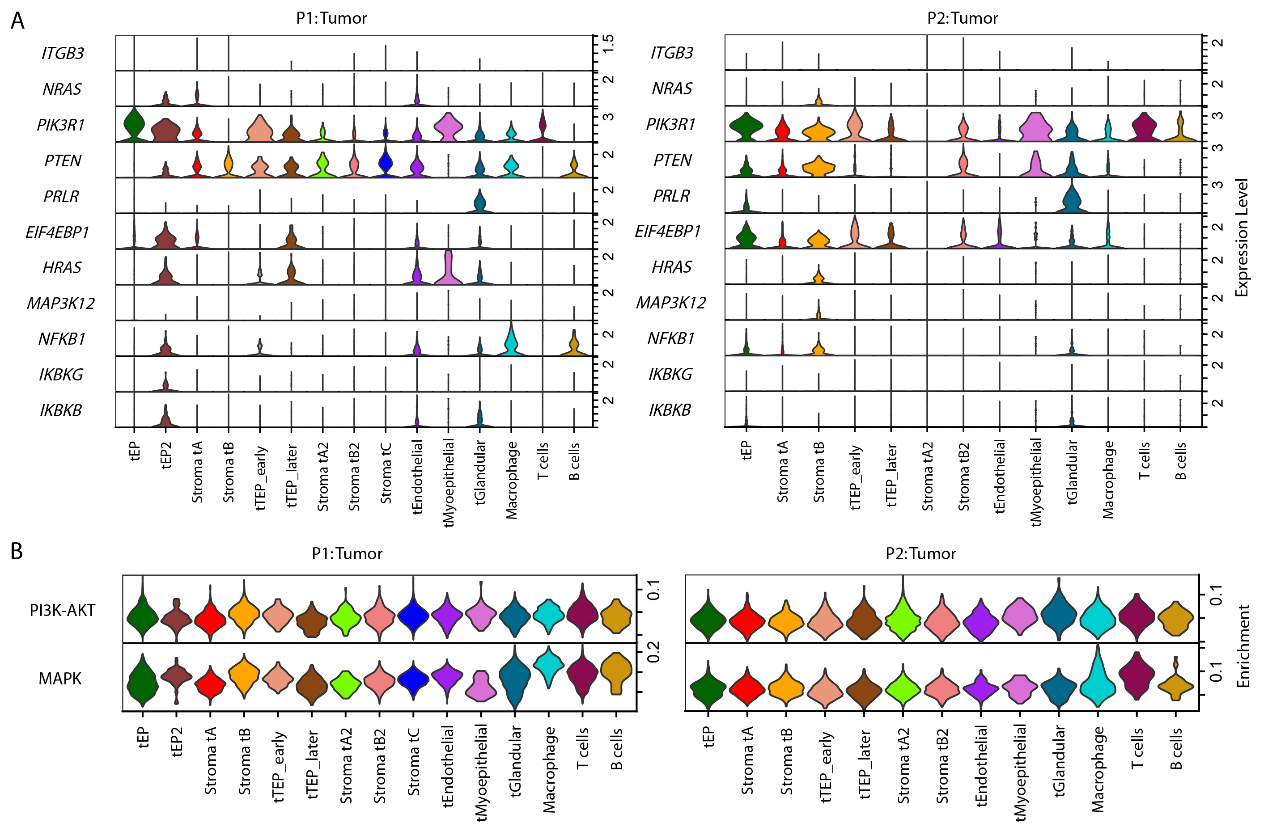


**Figure S3. Heterogeneous expression of PI3K-Akt and MAPK pathways in stromal cells.** (**A**) Violin distribution of expression levels of upregulated PI3K-Akt and MAPK pathway genes in malignant breast PTs as reported in a recent study. ^1^ (**B**) Enrichment scores of genes involved in PI3K-Akt and MAPK pathways in different cells, respectively.


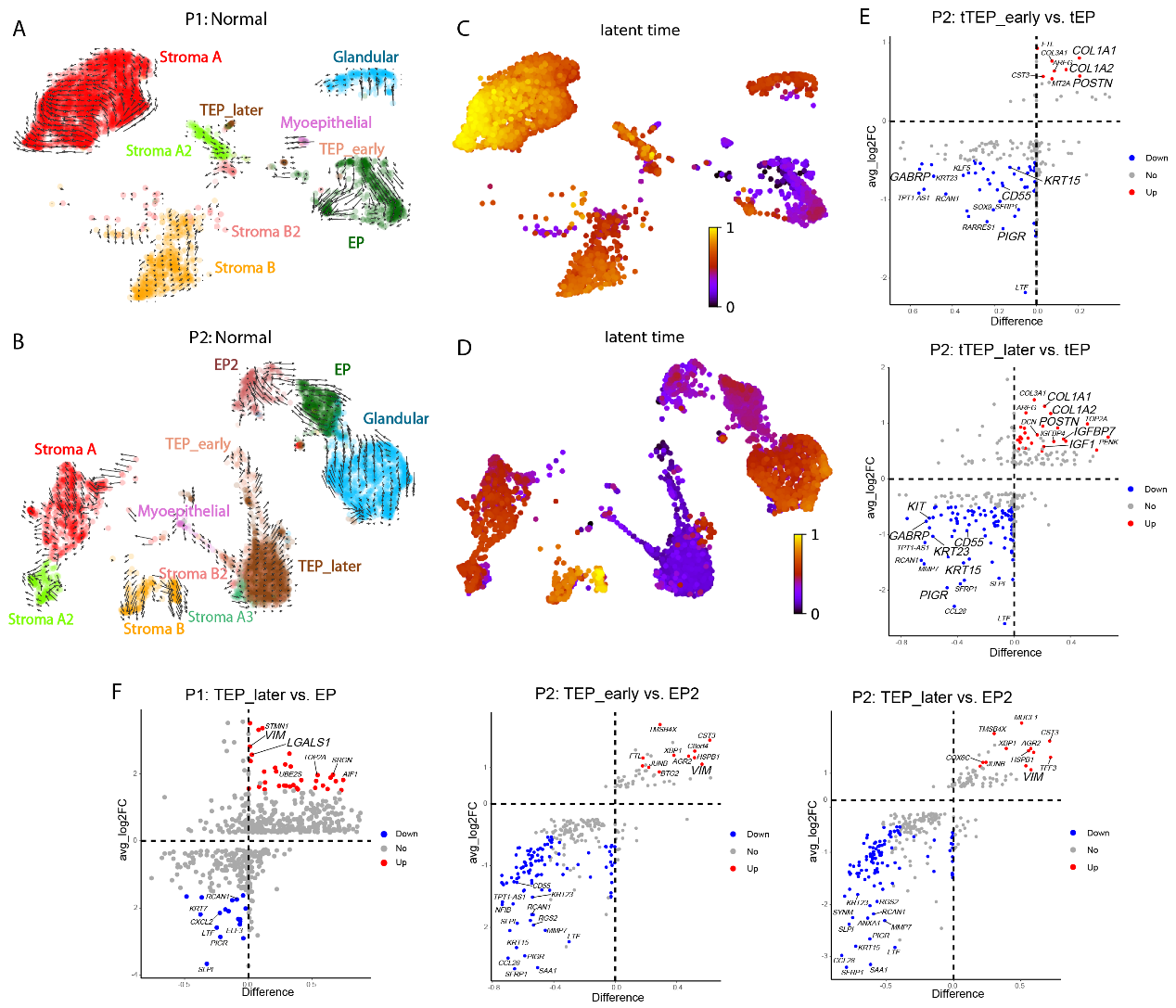


**Figure S4. Trajectory of stromal and epithelial cell lineage in adjacent normal tissues.** RNA velocity of stromal and epithelial cells in P1 (**A**) and P2 (**B**) normal tissues. The arrows represent the direction of transcriptional differentiation. Latent times of differentiation are shown in (**C**) and (**D**) accordingly. (**E–F**) Differential gene expression analysis between TEP and EPs in P2 tumor (**E**) and two patients’ adjacent normal tissues (**F**). The x-axis shows the difference in the proportion of cells where the gene is detected between the two groups. The y-axis shows the log fold-change of the average expression between the two groups.


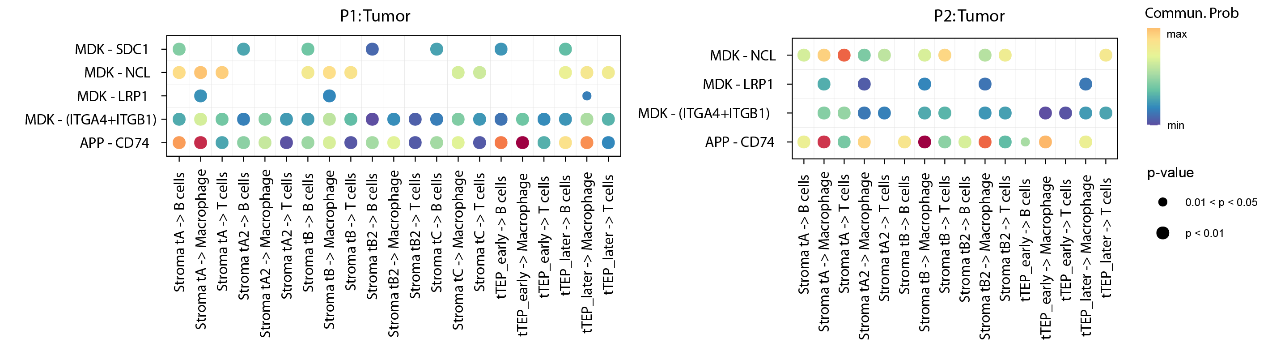


**Figure S5. Receptor-ligand pairs for stromal cell-immune cell communication.**


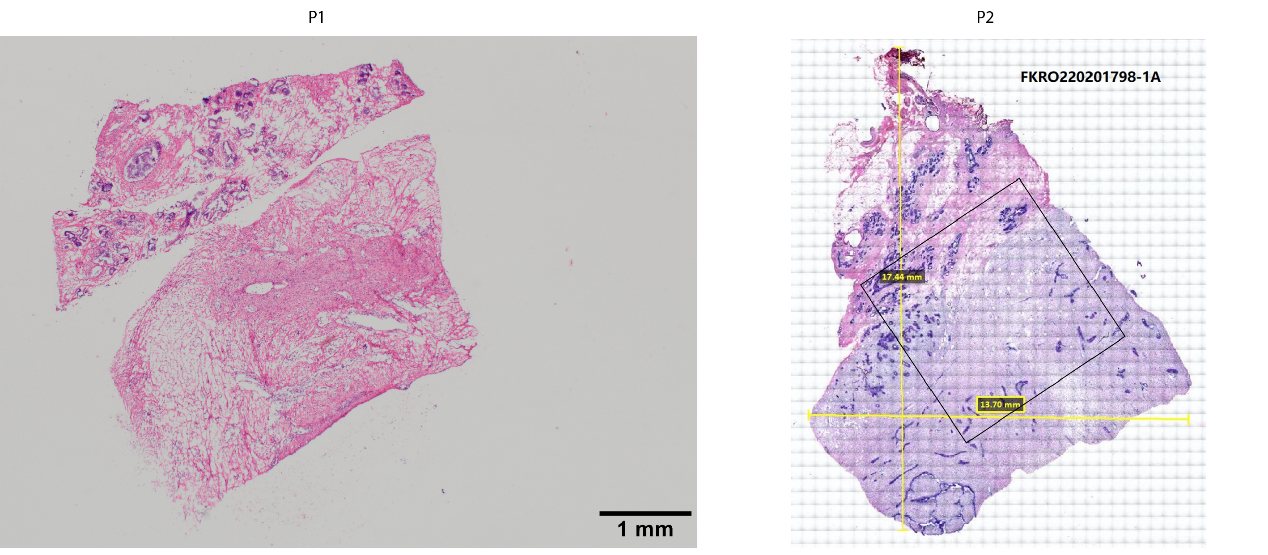


**Figure S6. H&E staining of P1 and P2 primary tissue sections for ST.**


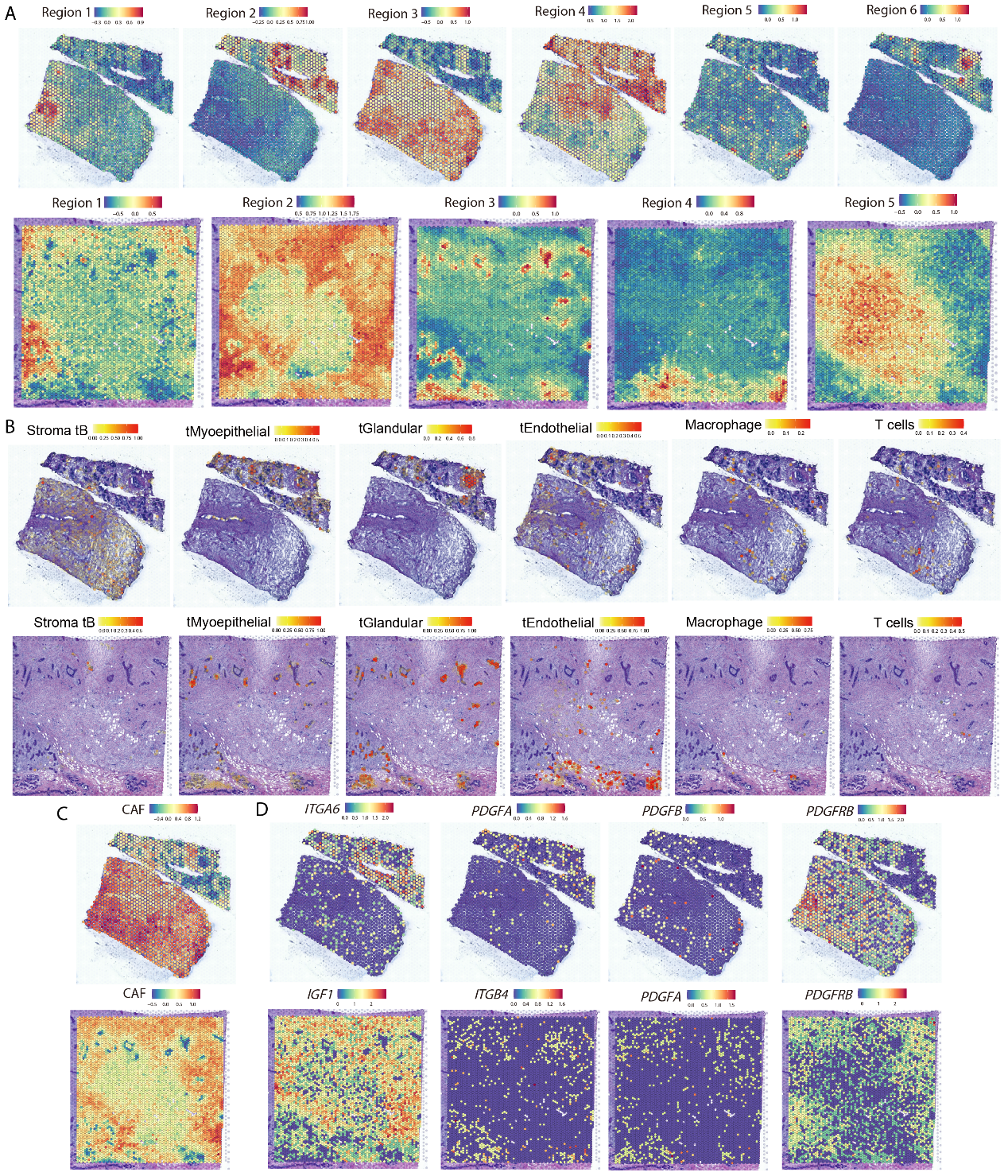


**Figure S7. Distributions of other cell types, enrichment of signature genes, and ligands and receptors on ST data.** (**A**) Enrichment of signature genes of each region on ST data of P1 (top) and P2 (bottom). (**B**) Distribution of other cell populations on ST data. (**C**) CAF enrichment distribution. (**D**) Expression distribution of other ligand-receptor pairs. The expression of *IGF1*, the ligand of Stroma tA, and integrin (*ITGA6* and *ITGB4*), the EP receptors, overlapped in the ST data. The expression of PDGF and its receptors in the ST also overlapped. In particular, *PDGFRB* was highly expressed in regions where tTEP_later cells were located, confirming the high expression of *PDGFRB* in differentiated stromal cells.


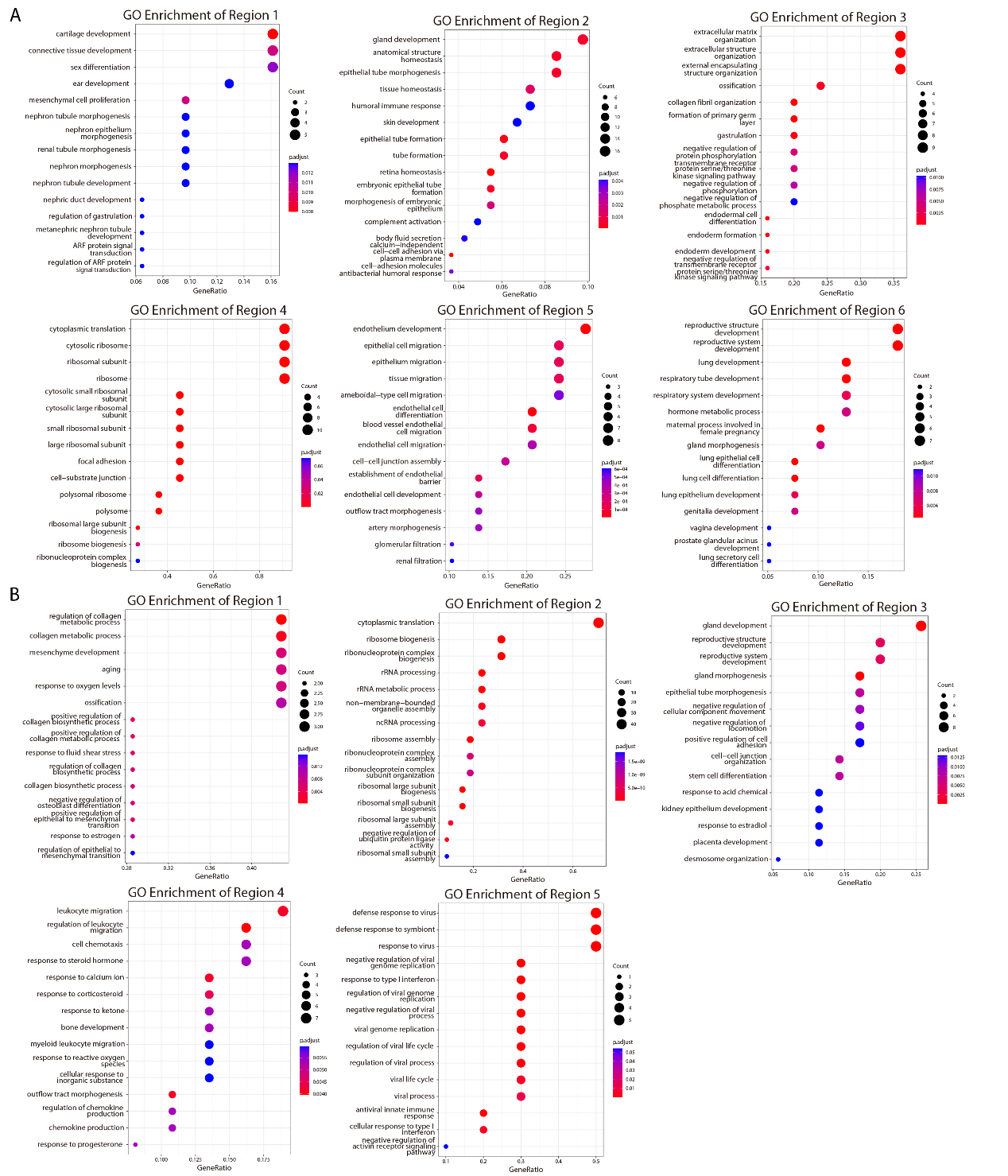


**Figure S8. GO functional enrichment of region signature genes in P1 (A) and P2 (B).**

**
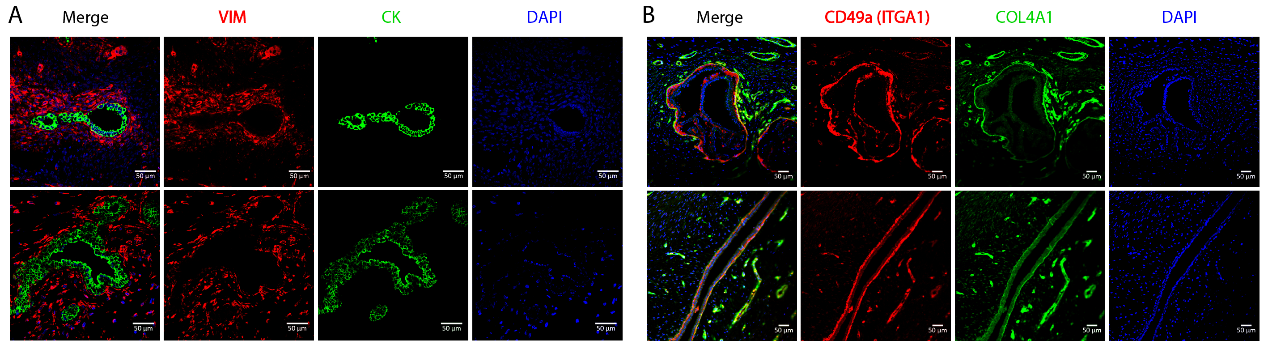
**

**Figure S9. Other representative IF staining.** (**A**) IF staining of VIM and CK in breast PT tumor tissues. (**B**) IF staining of ITGA1 and COL4A1 in borderline (top) and benign (bottom) breast PT tissue.

**
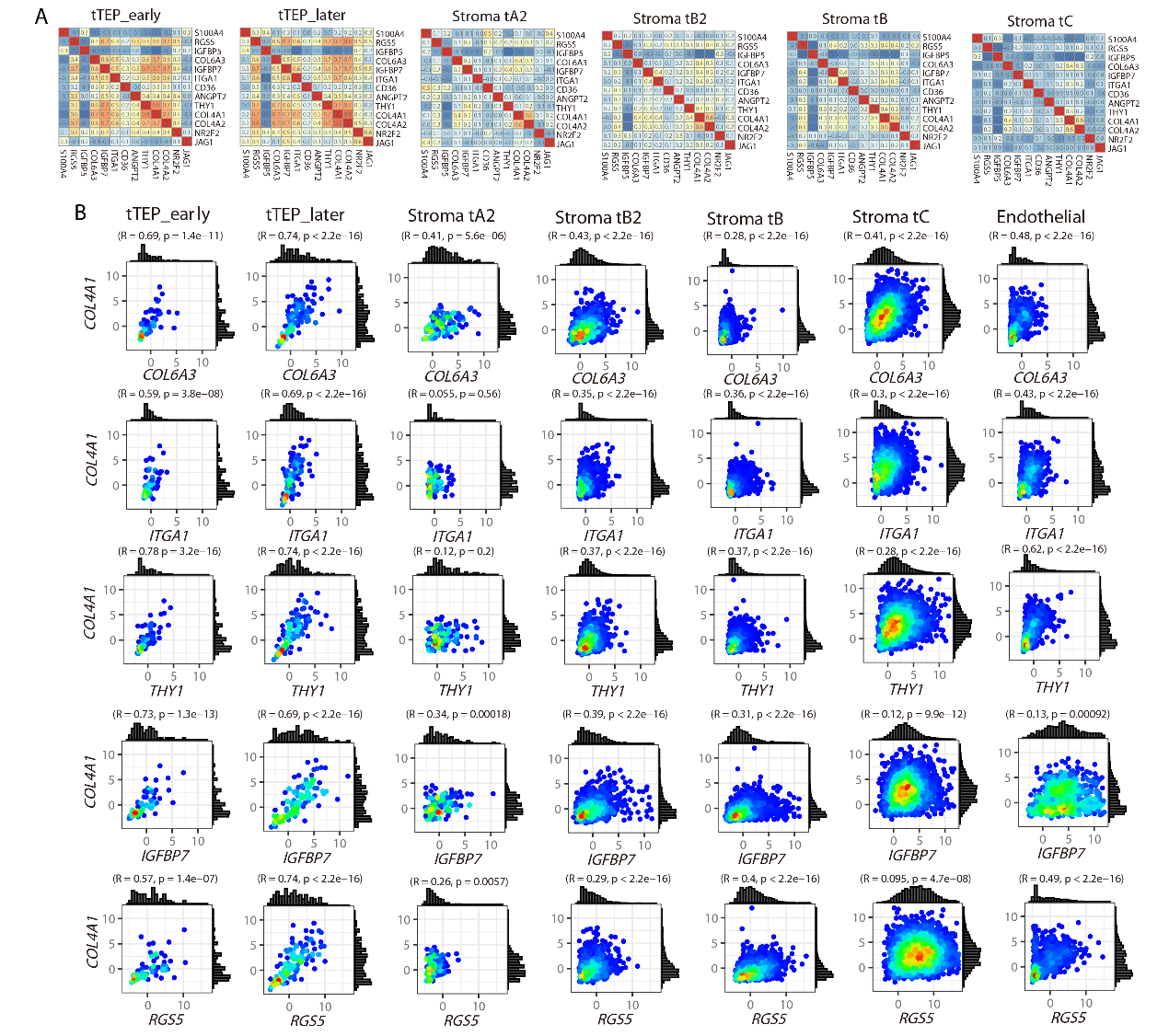
**

**Figure S10. Distribution of *COL4A1* expression with other genes in P1.** (**A**) Correlation of gene expression in differentiated stromal cells in P1. Numbers in the heatmap show Pearson correlation coefficients. (**B**) Distribution of *COL6A3*, *ITGA1*, *THY1*, *IGFBP7*, and *RGS5* expression with *COL4A1* in differentiated stromal and endothelial cells, respectively. Pearson correlation coefficients and p-values are shown above each scatterplot.


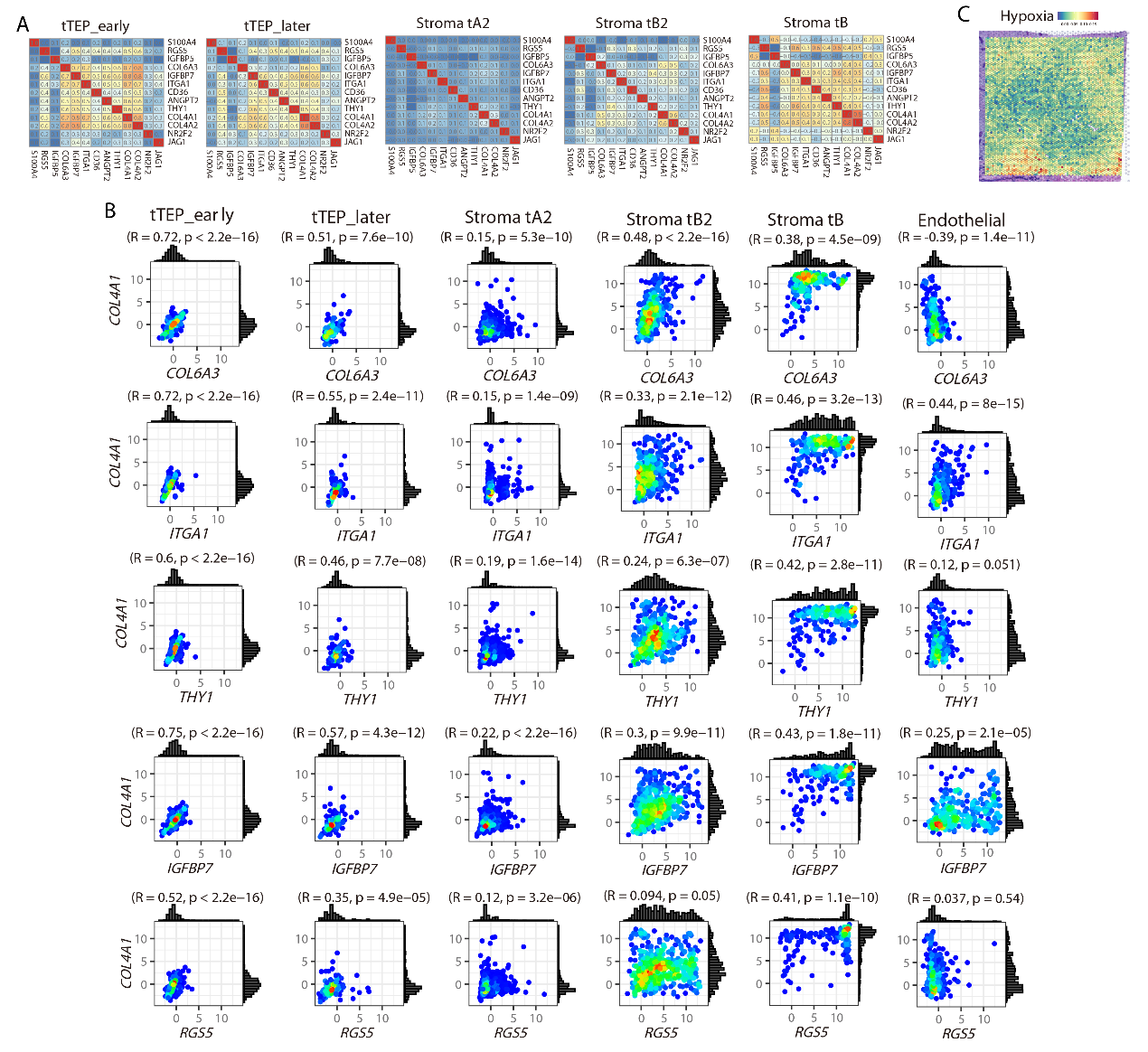


**Figure S11. Distribution of *COL4A1* expression with other genes in P2.** (**A**) Correlation of gene expression in differentiated stromal cells in P2. Numbers in the heatmap show Pearson correlation coefficients. (**B**) Distribution of *COL6A3*, *ITGA1*, *THY1*, *IGFBP7*, and *RGS5* expression with *COL4A1* in differentiated stromal and endothelial cells, respectively. Pearson correlation coefficients and p-values are shown above each scatterplot. (**C**) Enrichment of hypoxia signature in ST.


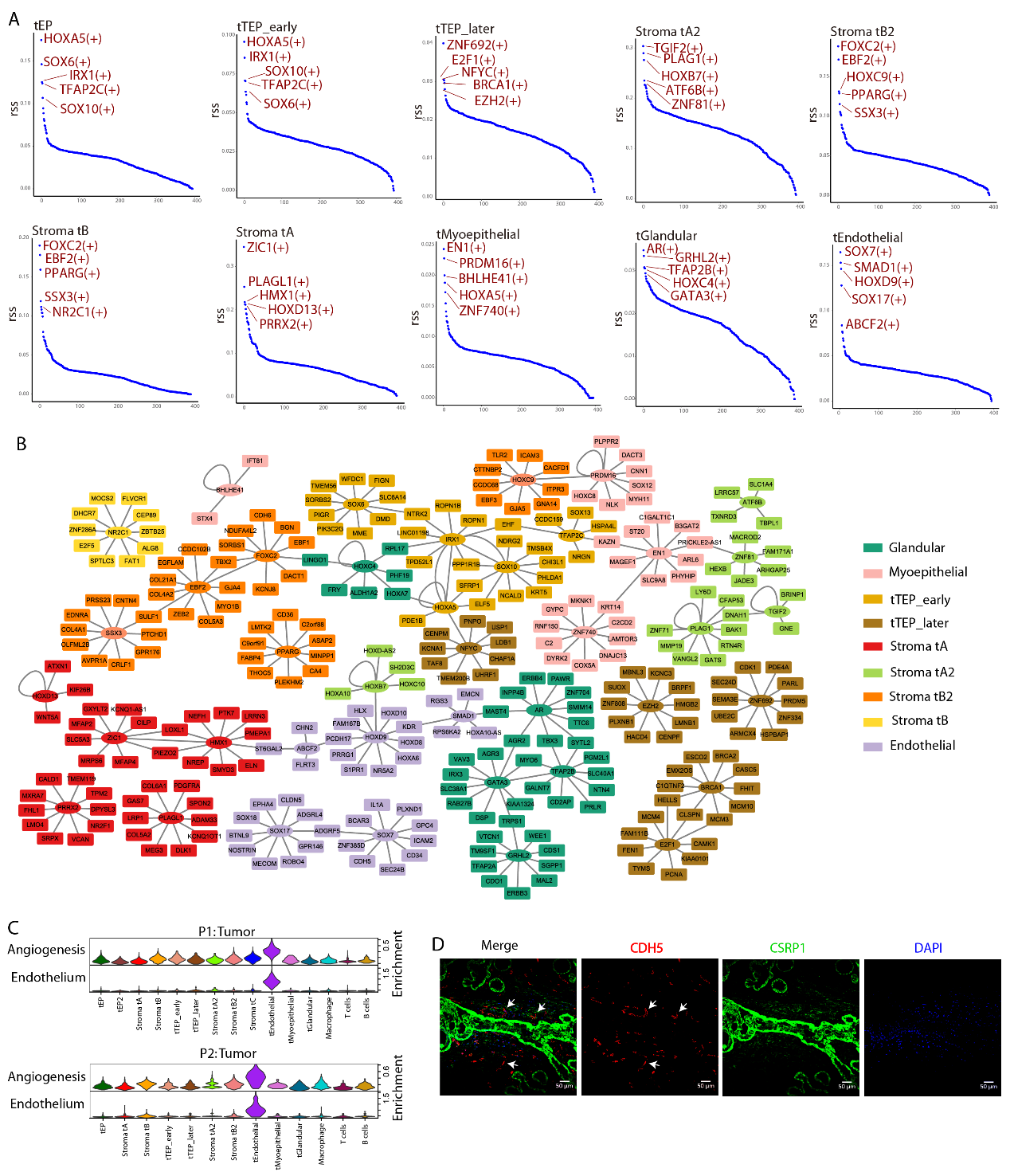


**Figure S12. Epithelial–stromal–endothelial intercellular regulatory relationships in P2.** (**A**) Epithelial, stromal, and endothelial cell-specific TFs in P2. (**B**) TF and target gene regulatory networks among epithelial-stromal-endothelial cells in P2. Different colors mark different cell populations. (**C**) Enrichment of signature genes of angiogenesis and endothelium in different cells. (**D**) IF staining shows the generation of microvessels in tumor tissue.


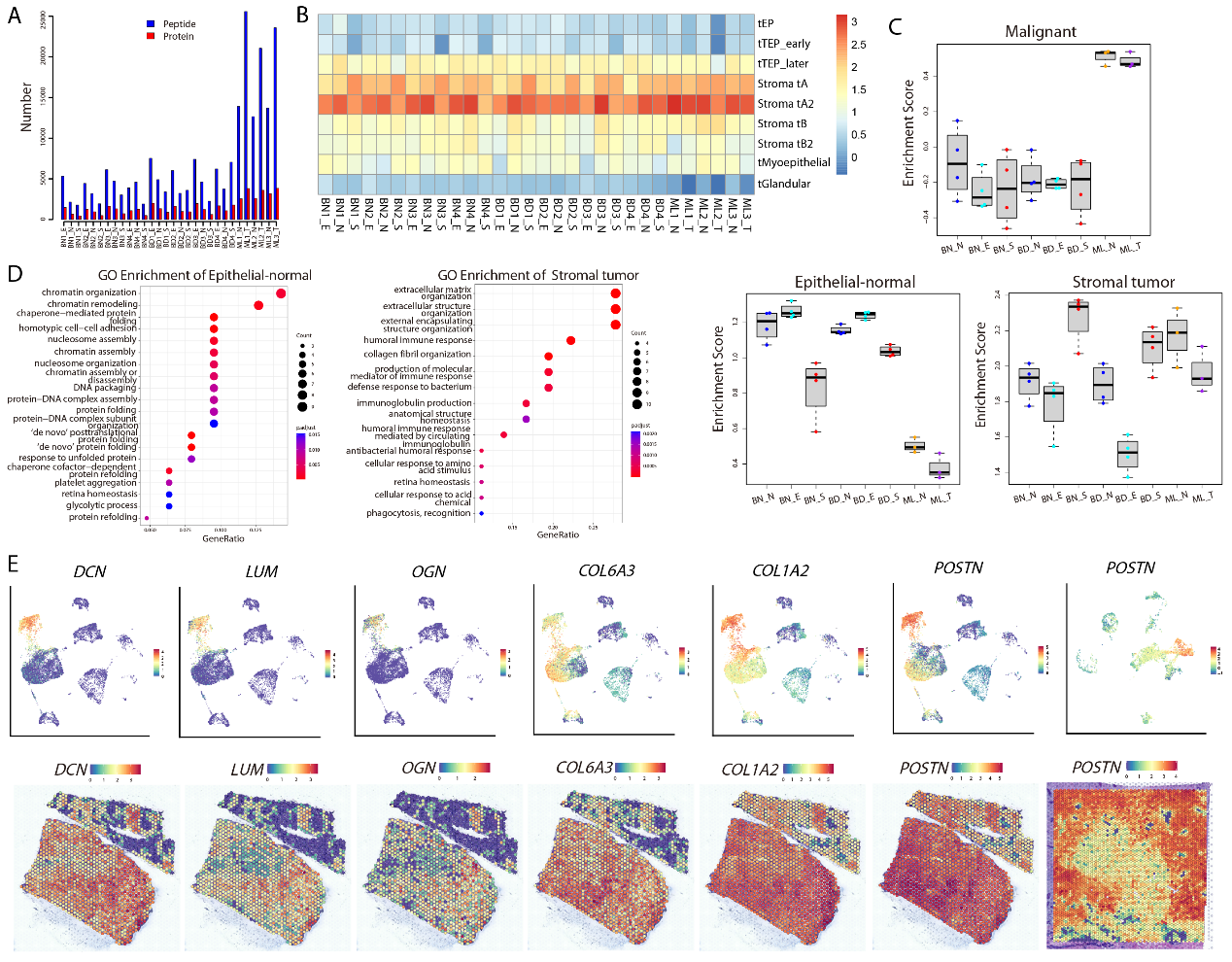


**Figure S13. Intratumoral heterogeneity versus inter-population heterogeneity.** (**A**) Distribution of the number of peptides and proteins captured in the samples. An average of 7,119 peptides and 1,586 proteins were captured. (**B**) Enrichment of signature genes of cell populations of P2 in the proteomic data. (**C**) Enrichment of each group’s signature proteins in different patients. (**D**) GO functional enrichment for group’s signatures. Functional analysis for the malignant group failed due to the small number of signatures obtained. (**E**) Representative examples of the distribution of signature gene expression in scRNA-seq and ST data.

**Supplementary tableS**

**Table S1. Clinical data of 97 breast PTs used for IHC validation.**

| case number | COL4A1 IHC score | COL4A2 IHC score | CSRP1 IHC score | age (years) | tumor size (mm) | location | tumor grade |
| --- | --- | --- | --- | --- | --- | --- | --- |
| 1 | 1+ | 0 | 0 | 36 | 30 | R | benign |
| 2 | 0 | 0 | 0 | 18 | 40 | L | benign |
| 3 | 0 | 0 | 0 | 21 | 23 | R | benign |
| 4 | 1+ | 2+ | 1+ | 49 | 35 | R | benign |
| 5 | 1+ | 0 | 1+ | 23 | 30 | R | benign |
| 6 | 0 | 0 | 1+ | 28 | 40 | R | benign |
| 7 | 1+ | 0 | 0 | 22 | 20 | R | benign |
| 8 | 1+ | 0 | 1+ | 38 | 10 | R | benign |
| 9 | 0 | 0 | 0 | 51 | 30 | R | benign |
| 10 | 1+ | 0 | 1+ | 24 | 27 | L | benign |
| 11 | 0 | 0 | 0 | 43 | 20 | L | benign |
| 12 | 0 | 0 | 1+ | 42 | 20 | L | benign |
| 13 | 0 | 0 | 0 | 28 | 22 | R | benign |
| 14 | 0 | 0 | 2+ | 27 | 25 | L | benign |
| 15 | 1+ | 0 | 1+ | 34 | 12 | R | benign |
| 16 | 1+ | 1+ | 1+ | 22 | 40 | L | benign |
| 17 | 0 | 0 | 2+ | 32 | 22 | R | benign |
| 18 | 0 | 0 | 0 | 20 | 12 | L | benign |
| 19 | 0 | 0 | 0 | 22 | 30 | L | benign |
| 20 | 0 | 0 | 0 | 24 | 22 | L | benign |
| 21 | 0 | 0 | 0 | 23 | 40 | R | benign |
| 22 | 1+ | 0 | 0 | 37 | 45 | R | benign |
| 23 | 1+ | 0 | 1+ | 28 | 25 | L | benign |
| 24 | 2+ | 1+ | 2+ | 43 | 25 | L | benign |
| 25 | 1+ | 0 | 0 | 18 | 40 | L | benign |
| 26 | 1+ | 1+ | 0 | 32 | 17 | R | benign |
| 27 | 1+ | 0 | 1+ | 22 | 24 | R | benign |
| 28 | 1+ | 0 | 0 | 32 | 18 | R | benign |
| 29 | 1+ | 0 | 0 | 31 | 15 | L | benign |
| 30 | 1+ | 1+ | 1+ | 43 | 25 | R | benign |
| 31 | 1+ | 1+ | 1+ | 23 | 25 | R | benign |
| 32 | 0 | 0 | 0 | 35 | 25 | R | benign |
| 33 | 3+ | 2+ | 0 | 42 | 43 | R | benign |
| 34 | 1+ | 0 | 0 | 43 | 28 | R | benign |
| 35 | 0 | 0 | 1+ | 29 | 32 | R | benign |
| 36 | 1+ | 0 | 0 | 26 | 27 | R | benign |
| 37 | 0 | 1+ | 0 | 32 | 34 | R | benign |
| 38 | 1+ | 0 | 0 | 40 | 32 | R | benign |
| 39 | 1+ | 1+ | 1+ | 35 | 26 | R | benign |
| 40 | 1+ | 0 | 0 | 28 | 39 | R | benign |
| 41 | 2 + | 0 | 0 | 27 | 35 | L | borderline |
| 42 | 3 + | 3 + | 0 | 38 | 18 | L | borderline |
| 43 | 1 + | 1 + | 1+ | 33 | 65 | R | borderline |
| 44 | 1 + | 0 | 2+ | 31 | 35 | R | borderline |
| 45 | 1 + | 0 | 1+ | 22 | 35 | L | borderline |
| 46 | 2 + | 1 + | 1+ | 36 | 27 | R | borderline |
| 47 | 1 + | 0 | 0 | 12 | 35 | L | borderline |
| 48 | 1 + | 0 | 1+ | 31 | 35 | L | borderline |
| 49 | 1 + | 1 + | 1+ | 33 | 27 | R | borderline |
| 50 | 1 + | 0 | 0 | 12 | 33 | R | borderline |
| 51 | 1 + | 0 | 1+ | 28 | 35 | R | borderline |
| 52 | 2 + | 0 | 0 | 15 | 55 | L | borderline |
| 53 | 1 + | 0 | 0 | 23 | 30 | L | borderline |
| 54 | 1 + | 2 + | 1 + | 29 | 30 | R | borderline |
| 55 | 0 | 0 | 0 | 27 | 35 | R | borderline |
| 56 | 0 | 1 + | 1 + | 44 | 35 | L | borderline |
| 57 | 1 + | 0 | 2 + | 38 | 70 | R | borderline |
| 58 | 0 | 0 | 1 + | 47 | 50 | R | borderline |
| 59 | 1 + | 0 | 2 + | 47 | 50 | L | borderline |
| 60 | 1 + | 1 + | 1 + | 38 | 30 | R | borderline |
| 61 | 0 | 0 | 2 + | 41 | 34 | R | borderline |
| 62 | 0 | 1 + | 1 + | 22 | 20 | L | borderline |
| 63 | 1 + | 1 + | 1 + | 56 | 45 | R | borderline |
| 64 | 0 | 0 | 0 | 43 | 20 | R | borderline |
| 65 | 2 + | 3 + | 1 + | 49 | 28 | L | borderline |
| 66 | 3 + | 2 + | 3 + | 34 | 38 | R | borderline |
| 67 | 3 + | 1 + | 1 + | 27 | 25 | R | borderline |
| 68 | 3 + | 1 + | 3 + | 40 | 30 | R | borderline |
| 69 | 3 + | 3 + | 3 + | 29 | 26 | L | borderline |
| 70 | 3 + | 2 + | 0 | 37 | 34 | R | borderline |
| 71 | 3 + | 3 + | 2 + | 33 | 26 | L | borderline |
| 72 | 1 + | 2 + | 2 + | 26 | 37 | R | borderline |
| 73 | 1 + | 0 | 0 | 42 | 20 | R | borderline |
| 74 | 2 + | 1 + | 1 + | 32 | 33 | R | borderline |
| 75 | 1 + | 0 | 1 + | 41 | 40 | L | borderline |
| 76 | 0 | 0 | 1 + | 26 | 36 | R | borderline |
| 77 | 0 | 1 + | 0 | 27 | 28 | R | borderline |
| 78 | 0 | 1 + | 0 | 32 | 43 | R | borderline |
| 79 | 1 + | 1 + | 2 + | 42 | 29 | R | borderline |
| 80 | 1 + | 1 + | 2 + | 38 | 31 | R | borderline |
| 81 | 2 + | 2 + | 0 | 19 | 10 | L | malignant |
| 82 | 2 + | 2 + | 2 + | 17 | 35 | L | malignant |
| 83 | 1 + | 1 + | 0 | 28 | 33 | R | malignant |
| 84 | 2 + | 3 + | 3 + | 35 | 28 | R | malignant |
| 85 | 1 + | 2 + | 0 | 26 | 30 | R | malignant |
| 86 | 0 | 1 + | 0 | 37 | 42 | R | malignant |
| 87 | 0 | 2 + | 2 + | 43 | 29 | R | malignant |
| 88 | 0 | 1 + | 0 | 48 | 37 | R | malignant |
| 89 | 3 + | 3 + | 2 + | 31 | 22 | L | malignant |
| 90 | 1 + | 2 + | 2 + | 47 | 50 | R | malignant |
| 91 | 0 | 2 + | 0 | 39 | 32 | R | malignant |
| 92 | 0 | 3 + | 2 + | 49 | 28 | L | malignant |
| 93 | 0 | 0 | 1 + | 45 | 30 | R | malignant |
| 94 | 3 + | 3 + | 0 | 32 | 45 | R | malignant |
| 95 | 1 + | 2 + | 0 | 40 | 36 | R | malignant |
| 96 | 1 + | 3 + | 0 | 36 | 23 | R | malignant |
| 97 | 2 + | 2 + | 0 | 38 | 35 | R | malignant |

**Table S2. Significance of the differences in positive staining for the three proteins between grades.**

| Comparison | COL4A1 | COL4A2 | CSRP1 |
| --- | --- | --- | --- |
| Benign vs. borderline | *P* = 0.025 | *P* = 0.01 | *P* = 0.037 |
| Benign vs. malignant | *P* = 0.02 | *P* < 0.001 | *P* = 0.007 |
| Borderline vs. malignant | *P* = 0.421 | *P* < 0.001 | *P* = 0.026 |

**Supplementary methods**

**Tissue dissociation**

The fat tissue and visible blood vessels were removed before tissue processing and washed with DMEM. We followed the “Dissociation of soft tumors” protocol from the Miltenyi tumor dissociation kit for dissociation. Briefly, the fully sheared tissues were placed in gentleMACS C tubes (Miltenyi Biotec, Auburn, CA, USA) containing 2.5 mL of tumor dissociation reagent, and incubated at 37 °C for 30 min. The cell suspension was passed through a 70 μm filter, and cells were separated by centrifugation at 500 ×*g* for 5 min. After initial processing, the cells were kept on ice for the remainder of the protocol. The samples were resuspended in red blood cell lysate on ice for 2 min, centrifuged at 300 ×*g* for 5 min, and washed with DMEM two times. Freshly prepared cells were suspended in the DMEM buffer at the requried concentration.

**Sample preparation and optimization for ST**

Fresh tumor tissues and adjacent normal tissues from P1 and P2 were collected, frozen, and embedded in optical cutting tissue compounds using liquid nitrogen. The RNA quality of the optical cutting tissue-embedded blocks was assessed using an Agilent 2100 (Agilent Technologies, Waldbronn, Germany). Only tissues with an RNA integrity number greater than seven were used for the visible spatial gene expression experiment. Cryosections were obtained on a Leica CM3050S (Leica Biosystems Nussloch GmbH, Heidelberger, Germany), and bright-field images were captured on a Leica Aperio Versa8 whole-slide scanner (Leica Microsystems CMS GmbH, Heidelberger, Germany) at 20x resolution.

The Visium spatial tissue optimization slide and reagent kit (10x Genomics) were used to optimize the permeabilization conditions for the tissues according to the manufacturer’s user guide (CG000238, 10x Genomics). In summary, the workflow included mounting the tissue sections on Visium tissue optimization slides (1000192, 10x Genomics). Sections were fixed, stained with H&E, and permeabilized for different periods. The mRNAs released during permeabilization bound to oligonucleotides in the capture areas. Fluorescent cDNAs were synthesized on slides and imaged. The permeabilization time that resulted in the maximum fluorescence signal and the lowest signal diffusion was optimal. If the signals were the same at both time points, a longer permeabilization time was considered optimal.

**scRNA-seq data filtration, quality control, and normalization**

Based on the count matrix, we used the R package DoubletFinder (RRID: SCR_018771,

version 2.0.3)^2^ to predict putative cell doublets and applied the SoupX (version 1.6.1)^3^ R package to remove ambient contaminating RNA. Quality control and normalization were performed using the R package Seurat (version 4.1.1)^4^. Cells containing fewer than 500 genes and more than 20% of the reads mapped to the mitochondrial genome were excluded. After stringent quality control, 35,431 cells were retained for subsequent analysis, of which 12,235 cells originated from tumors 9,920 cells from adjacent normal tissues in P1, 5,076 cells from tumors, and 8,200 cells from normal tissue in P2. To do further analysis, we need to normalize the data. Count data were log-normalized using the default NormalizeData function in Seurat, followed by the default ScaleData function by converting normalized gene expression to Z-score (values centered at 0 and with variance of 1). We then performed a principal component analysis on the scaled expression values using the 2,000 most variable genes by the RunPCA function. Cells were clustered using the first 20 principal components as inputs in the FindNeighbors function in Seurat. Once clustering was done, we visually and carefully examined the dependence of mitochondrial RNA, ribosomal RNA, and cell cycle genes on cell clusters using the RunUMAP, DimPlot, FeaturePlot, and VlnPlot functions. After this extensive quality control, we renormalized with the SCTransform function in Seurat by regressing these factors using vars.to.regress variables. Since SCTransform replaces NormalizeData and ScaleData, the normalization step of SCTransform can be done using the RNA assay which stored the count matrix of the Seurat object. The normalized values were stored in the SCT assay which can be easily switched due to different downstream analysis purposes.

**Identifying cell clusters using the NMF method**

The top 5,000 variable genes were identified using the Seurat FindVariableFeatures function, followed by scaling and centering using the ScaleData function. Clustering was performed using the R NMF package (version 0.24.0)^5^ at different ranks (2–10). A total of 10 runs were performed for each rank to obtain consensus clustering. To determine the number of cell clusters, the distribution of cell marker gene expression was visualized using the DimPlot function to obtain the overall structure of cell types. Subsequently, different ranks were set to classify the cells according to their types, and the optimal rank was determined by aligning clusters with known marker genes expression for cell types. For a given optimal rank, we ran the nmf function with the parameter "n = 10, seed = random" for several times, until the classification of cell subpopulations was stable. It should be noted that the NMF algorithm also estimates an optimal rank which can be chosen by plotting the cophenetic coefficient values. We also considered the cophenetic plot as it suggested the underlying subpopulation compositional structure of cell populations. Indeed, we found that the heterogeneity of marker gene expression in the same cell type can be well represented by the delineated subpopulations. Signature genes for each cluster were extracted using the extractFeatures function in NMF, based on their contributions to each cluster. Non-linear dimensionality reduction was performed by running the runUMAP function in Seurat and then used for cell cluster visualization.

**Cell identity determination**

Each NMF cluster was annotated by their expression of known cell marker genes, including myoepithelial cells (*EPCAM*, *KRT5*, *KRT14*, *ITGA6*, and *ACTA2*), glandular cells (*EPCAM*, *KRT8*, *KRT18*, *KRT19*, and *GATA3*), EPs (*EPCAM*, *CD24*, *CD44*, *KIT*, *GABRP*, and *CD55*), stromal cells (*LUM*, *FBLN1*, *COL1A1*, *COL1A2*, *COL3A1*, *COL5A1*, *ACTA2*, *TAGLN*, and *COL6A3*), endothelial cells (*VWF* and *CDH5*), T cells (*CD2*, *CD3D*, *CD3E*, and *CD8A*), macrophages (*CD86*, *CD68*, and *CSF1R*), and B cells (*MS4A1*, *CD79A*, and *CD79B*). In stromal cells, *LUM*, *FBLN1*, *COL1A1*, *COL1A2*, *COL3A1*, *COL5A1*, and *COL6A3* were used to mark fibroblasts and *ACTA2*, and *TAGLN* to distinguish myofibroblasts.

**Trajectory reconstruction and identification of genes with dominant expression changes**

The analysis utilized scVelo (version 0.2.4)^6^ to reconstruct the trajectories and identify cell-state transitions. scVelo analyzed the expression dynamics by estimating the RNA velocities of single cells and distinguishing between unspliced and spliced transcripts. Using the sorted cell ranger-generated BAM files, we generated loom files of the scRNA-seq data by running a velocyto (version 0.17.17)^7^ pipeline. The meta data and cell embedding information were extracted from the Seurat object, serving as inputs for scVelo. The analysis used a dynamic model of scVelo, and the latent time of cellular occurrence was calculated accordingly. Genes with the highest expression variation among cells were sorted based on their likelihood values. We manually examined the top 200 genes using high likelihood in the dynamic model, and genes that displayed pronounced dynamic behavior along with latent time were selected.

**Cell-cell communication analysis**

The CellChat package (version 1.1.3)^8^ was used to infer the communication between cells and visualize intercellular communication networks from the scRNA-seq data. CellChat integrates single-cell gene expression with prior knowledge of the interactions between signaling ligands, receptors and their cofactors. First, a single-cell count matrix with cell types as labels, was input into CellChat. Using these labels, CellChat identified differentially overexpressed ligands and receptors for each cell group using the Wilcoxon rank sum test with a significance level of 0.05. Communication probability between two cell groups was quantified by the law of mass action model. The significance of intercellular communications was identified using a permutation test with a significance level of *P* < 0.05 considered significant. A minimum of 10 cells was considered in each cell group for cell-cell communication. We summarized the probabilities of all interactions associated with each signaling pathway and compared tumor and normal samples. We found that the COLLAGEN pathway occurred prominently in the tumor samples; thus, we studied the detailed ligand–receptor pairs in this pathway. In addition, based on the growth factors identified in the scRNA-seq data, we carefully analyzed the receptor-ligand pair interactions of the PDGF, IGF, WNT, and NOTCH pathways.

**Transcriptional regulatory relationship analysis**

To infer the gene regulatory network, we applied the SCENIC^9^ workflow to the normalized expression matrix. Considering the computation speed, we first run a command line of pySCENIC (version 0.11.2) following three steps: “grn,” “ctx,” and “aucell.” The Python version used was 3.7.13. SCENIC calculates the regulon specificity score (rss) to identify cell subpopulation-specific regulons. SCENIC searched for genes co-expressed with TFs by analyzing cis-regulatory motifs and identifying putative direct-binding targets. Only the significantly enriched motifs of the correct upstream regulator were retained and pruned to remove indirect target genes without motif support. Thus, the target genes could be enriched in TF-binding motifs. A regulon is comprised of a set of TF and their potential target genes. We extracted the top five most specific TFs with the highest regulon specificity scores and the top ten target genes for each TF from the SCENIC results. Based on the regulatory relationships between these TFs and their target genes, we constructed regulatory networks among different cell populations using Cytoscape (RRID: SCR_003032, version 3.9.1)^10^.

**Extraction of proteins and peptides derived from the LCM tissues**

LCM tissues were dewaxed in xylene twice for 5 min and washed at 37 °C in 100%, 80%, and 60% ethanol for 5 min. The cells were then extracted and suspended in an appropriate amount of 1x Cocktail with EDTA and without SDS L3 (7 M urea, 2 M thiourea, and 20 mM TRIS-HCl, pH 8.0), and DTT was added at a final concentration of 10 mM. Cells were lysed followed by ultrasonication (VCX130, Sonics Inc, Newtown, CT, USA) and centrifugation at 25,000 ×*g* at 4 °C for 15 min. The suspension was reduced with 10 mM DTT for 1 h, alkylated with 55 mM IAM for 45 min, and diluted with 50 mM NH_4_HCO_3_ for protein digestion. A total of 2.5 μg of trypsin enzyme was added in the ratio of protein:enzyme at 40:1 and digested for 4 h at 37 °C. Enzymatic peptides were desalted using a Strata X column and vacuumed to dryness. Equal amounts of peptides were extracted from all samples, mixed, diluted with mobile phase A (5% ACN pH 9.8), and injected. The Shimadzu LC-20AB HPLC system coupled with a Gemini high pH C18 column (5 μm, 4.6 × 250 mm) was used. The sample was subjected to column chromatography and then eluted at a flow rate of 1 mL/min using the following gradient: 5% mobile Phase B (95% CAN, pH 9.8) for 10 min, 5% to 35% mobile phase B for 40 min, 35% to 95% mobile phase B for 1 min, flow phase B lasted 3 min, and 5% mobile phase B was equilibrated for 10 min. The elution peak was monitored at a wavelength of 214 nm, and the components were collected every minute. The components were combined into 10 fractions, which were freeze-dried.

**Generation of microproteomic data**

The dried peptide samples were reconstituted with mobile phase A (2% ACN and 0.1% FA), centrifuged at 20,000 ×*g* for 10 min, and the supernatant was collected for injection. The separation was performed using a Thermo UltiMate 3,000 UHPLC liquid chromatograph. The sample was first enriched in the trap column and desalted, entered a tandem self-packed C18 column (150 μm internal diameter, 1.8 μm column size, 35 cm column length), and separated at a flow rate of 500 nL/min. Each peptide fraction was separated and ionized by nanoESI and injected into tandem mass spectrometer Orbitrap Fusion™ Lumos™ Tribrid™ Mass Spectrometer (Thermo Fisher Scientific, San Jose, CA, USA) with DDA (data-dependent acquisition) detection mode. For DIA (data-independent acquisition) analysis, LC-separated peptides were ionized by nanoESI and injected into tandem mass spectrometer Orbitrap Fusion™ Lumos™ Tribrid™ mass spectrometer (Thermo Fisher Scientific, San Jose, CA, USA) with DIA detection mode. The main settings were as follows: ion source voltage, 2 kV; MS scan range, 400–1,500 m/z; MS resolution, 60,000; MIT, 50 ms; and 400–1,500 m/z were equally divided into 44 continuous window MS/MS scans. MS/MS collision-type HCD, MIT 54 ms. Fragment ions were scanned using Orbitrap, MS/MS resolution 30,000, and collision energy 30; AGC was 5E4.

**IHC and IF staining**

IHC experiments were performed using the LEICA immunohistochemical staining platform (LEICA, Bond-III, Leica Biosystems Nussloch GmbH, Heidelberger, Germany). Formalin fixed and paraffin embedding tissue was performed to paraffin sections with 3-μm thickness. Conventional dewaxing in double-distilled water, antigen repair with boiling EDTA at pH 9.0 for 20 min, cooling to 37 °C, and washing with phosphate-buffered saline (PBS) three times for 5 min each. The sections were blocked with 1% bovine serum albumin in PBS for 15 min. Further, sections were incubated with the following antibodies: rabbit anti-CSRP1 (orb48990, Biorbyt, UK), mouse anti-VE-cadherin/CDH5 (GeneTex Cat# GTX633705, RRID: AB_2888370, GeneTex, USA), mouse anti-collagen Type IV α2 chain /COL4A2 (MAB1910, MilliporeSigma, USA), rabbit anti-collagen type IV α1 chain/COL4A1 (NBP3-12908, Novus Biologicals, USA), mouse anti-Integrin Alpha 1/CD49a/ITGA1 (NBP2-76478, Novus Biologicals, USA), rabbit anti-CK (RAB-0050, MXB Biotechnologies, China), rabbit anti-CD117/KIT (Kit-0029, MXB Biotechnologies, China), mouse anti-vimentin/VIM (Kit-0019, MXB Biotechnologies, China), and mouse anti-CD34 (Kit-0004, MXB Biotechnologies, China) antibodies overnight at 4 °C. After rinsing with PBS three times for 5 min each, Tregs were incubated with the secondary antibody for 15 min at 37 °C and then rinsed three times with PBS.

For IF staining, Tregs were incubated with fluorescein isothiocyanate-labeled goat anti-mouse IgG (Abcam Cat# ab150116, RRID: AB_2650601, Abcam, UK) and goat anti-rabbit IgG (Abcam Cat# ab150077, RRID: AB_2630356, Abcam, UK) as a secondary antibody for 1 h at 37 °C. The sections were washed and counterstained with nuclear dye 4,6-diamino-2-phenylindole. Images were captured using a confocal microscope (LSM710, Zeiss, Germany).

IHC scoring for COL4A1, COL4A2, and CSRP1 was performed separately by two trained pathologists without knowledge of the case outcomes. Samples were scored as 0 if negative staining; 1+ when 10% or less of the cells showed weak, barely detectable, incomplete cytoplasmic or nuclear staining; 2+ when at least 10% of the cells showed intact, weak to moderate cytoplasmic or nuclear staining; and 3+ when at least 10% of the cells showed circumferential, intact, intense cytoplasmic or nuclear staining. For illustration, we defined 1+ as low expression and combined 2+ and 3+ as high expression.

**TEM operation**

1 mm^3^ of tissue was placed in pre-chilled 2.5% glutaraldehyde solution and fixed at 4 °C for 12 h. After washing the tissue three times with 0.1 mol phosphate buffer, it was fixed with 1% osmium for 1 h at 20–25 °C. The tissues were then soaked with resin mixed with propylene oxide (1:1) for 2 h at 20–25 °C, followed by saturation with pure resin for 12 h. The tissue was then embedded in fresh pure resin and polymerized at 45 °C for 12 h. Next, the tissue was cut into semithin sections of 1-μm thickness using an ultrathin sectioning machine. The sections were stained with toluidine blue, viewed under a microscope at 1 or 2 μm resolution, and selected for preparation of ultrathin sections. Ultrathin sections were prepared using an ultrathin-sectioning machine and transferred to a 200-mesh copper mesh. Ultrathin sections of the copper mesh were fixed onto stained silicone plates and coated with 3% lead citrate, 5% uranyl acetate, and 3% lead citrate. After drying the copper mesh at 20–25 °C, the morphological characteristics of the tumor stromal cells were observed by TEM (JEM-1400, JEOL, Tokyo, Japan).

**Primary human breast PTs tumor cell culture and immortalized cell establishment**

Tumor tissues from a borderline breast PT were washed three times with PBS and divided into multiple 1 mm^3^ tissue blocks. Further, 0.25% trypsin-EDTA (Gibco, USA) was added and digested for 1 h at 37 ℃. The digested cell suspension was filtered through a 70-μm cell strainer (Sorfa, China) and centrifuged at 1000 rpm and 300 ×*g* for 5 min. After centrifugation, the supernatant was discarded, and the cells were resuspended in 25-cm culture flasks with Advanced DMEM/F-12 (Gibco, USA) containing 10% FBS (Gibco, USA) and 100 U/mL penicillin-streptomycin (Gibco, USA). The cells were incubated at 37 °C in a humidified incubator containing 5% CO_2_ and allowed to grow against the wall. The complete medium was changed every 2 days until the cells covered 85–90% of the culture dish. SV40T lentiviral infection of primary PT cells allowed them to acquire immortalization abilities.

**Supplementary contents**

**Correlation between expression of *COL4A1/2* and genes with dominant expression changes**

By examining changes in gene expression with latent time, we found that *COL4A1*, *IGFBP7*, and *RGS5* governed the progression of differentiated stromal cells in both patients. Furthermore, we noticed that the expression of *COL4A1/2*, *THY1*, *ITGA1*, *IGFBP7*, *COL6A3*, and *RGS5* was well correlated in TEP and malignant Stroma tB cells but not in differentiated stromal cells (**Figure S10A, S11A**). Thus, we carefully examined the expression of other genes with respect to the distribution of *COL4A1* (**Figure S10B, S11B**). The expression of *COL6A3*, *ITGA1*, and *THY1* in P1 displayed a right-skewed distribution, indicating that their expression increased in cells at a rate lagging behind that of *COL4A1*. Even in P2, they did not increase their expression in cells to the same extent as *COL4A1*. In contrast, the expression levels of *RGS5* and *IGFBP7* in the cells increased, similar to that of *COL4A1*. Cells with high expression of *COL4A1* also highly expressed *RGS5* and *IGFBP7*. Notably, *IGFBP7* was predominantly expressed in endothelial cells. We speculated that the release of large amounts of *IGFBP7* from endothelial cells may provide a growth advantage for stromal cells. While *RGS5* is a hypoxia-induced gene, ^11^ its persistently high expression in stromal cells may be a response to the hypoxic environment of tumors (**Figure S11C**).

**Regulatory relationships between stromal cells and endothelial cells**

The regulatory network showed that stromal cells and endothelial cells maintained a tightly regulated relationship with each other (**Figures 7B and S12B**). For example, while HES5 acts as a TF in Stroma tC cells, *HES5* is also a target gene of HEY1 in endothelial cells. Its target genes include *PDGFB* and *ANGPT2*, which were expressed in both Stroma tC and endothelial cells. Moreover, angiogenesis signature genes were enriched in endothelial cells (**Figure S12C**). IF staining revealed the generation of microvessels near the glands (**Figure S12D**). Thus, endothelial cells play a role in maintaining transcriptional regulatory relationships with stromal cells and forming blood vessels to provide essential oxygen and nutrients to highly active stromal cells.

**Supplementary references**

1 Li X, Vail E, Maluf H, et al. Gene Expression Profiling of Fibroepithelial Lesions of the Breast. *Int J Mol Sci.* 2023; 24(10).

2 McGinnis CS, Murrow LM, Gartner ZJ. DoubletFinder: Doublet Detection in Single-Cell RNA Sequencing Data Using Artificial Nearest Neighbors. *Cell Syst.* 2019; 8(4): 329-337.e324.

3 Young MD, Behjati S. SoupX removes ambient RNA contamination from droplet-based single-cell RNA sequencing data. *Gigascience.* 2020; 9(12).

4 Hao Y, Hao S, Andersen-Nissen E, et al. Integrated analysis of multimodal single-cell data. *Cell.* 2021; 184(13): 3573-3587 e3529.

5 Gaujoux R, Seoighe C. A flexible R package for nonnegative matrix factorization. *BMC Bioinformatics.* 2010; 11: 367.

6 Bergen V, Lange M, Peidli S, Wolf FA, Theis FJ. Generalizing RNA velocity to transient cell states through dynamical modeling. *Nat Biotechnol.* 2020; 38(12): 1408-1414.

7 La Manno G, Soldatov R, Zeisel A, et al. RNA velocity of single cells. *Nature.* 2018; 560(7719): 494-498.

8 Jin S, Guerrero-Juarez CF, Zhang L, et al. Inference and analysis of cell-cell communication using CellChat. *Nat Commun.* 2021; 12(1): 1088.

9 Aibar S, Gonzalez-Blas CB, Moerman T, et al. SCENIC: single-cell regulatory network inference and clustering. *Nat Methods.* 2017; 14(11): 1083-1086.

10 Shannon P, Markiel A, Ozier O, et al. Cytoscape: a software environment for integrated models of biomolecular interaction networks. *Genome Res.* 2003; 13(11): 2498-2504.

11 Jin Y, An X, Ye Z, et al. RGS5, a hypoxia-inducible apoptotic stimulator in endothelial cells. *J Biol Chem.* 2009; 284(35): 23436-23443.
